# ECSIT‐X4 is Required for Preventing Pressure Overload‐Induced Cardiac Hypertrophy via Regulating Mitochondrial STAT3

**DOI:** 10.1002/advs.202414358

**Published:** 2025-01-02

**Authors:** Xia Lu, Tingting Tong, Haoliang Sun, Yi Chen, Yongfeng Shao, Pengxi Shi, Linli Que, Li Liu, Guoqing Zhu, Qi Chen, Chuanfu Li, Jiantao Li, Shuo Yang, Yuehua Li

**Affiliations:** ^1^ Key Laboratory of Targeted Intervention of Cardiovascular Disease Collaborative Innovation Center for Cardiovascular Disease Translational Medicine School of Basic Medical Sciences Nanjing Medical University Nanjing Jiangsu 211166 China; ^2^ Department of Cardiology Shanghai Sixth People's Hospital Affiliated to Shanghai Jiao Tong University School of Medicine Shanghai 200233 China; ^3^ Department of Cardiovascular Surgery The First Affiliated Hospital of Nanjing Medical University Nanjing Jiangsu 210029 China; ^4^ Department of Geriatrics the First Affiliated Hospital of Nanjing Medical University Nanjing Jiangsu 210029 China; ^5^ Department of Surgery East Tennessee State University Campus Box 70575 Johnson City TN 37614‐0575 USA; ^6^ Department of Immunology Key Laboratory of Immunological Environment and Disease State Key Laboratory of Reproductive Medicine Nanjing Medical University Nanjing Jiangsu 211166 China

**Keywords:** cardiac hypertrophy, ECSIT‐X4, mitochondria, STAT3

## Abstract

Mitochondrial dysfunction is a key factor in exacerbating pressure overload‐induced cardiac hypertrophy and is linked to increased morbidity and mortality. ECSIT, a crucial adaptor for inflammation and mitochondrial function, has been reported to express multiple transcripts in various species and tissues, leading to distinct protein isoforms with diverse subcellular localizations and functions. However, whether an unknown ECSIT isoform exists in cardiac cells and its potential role in regulating mitochondrial function and pathological cardiac hypertrophy has remained unclear. This study identified a 42‐kDa ECSIT isoform encoded by the transcript variant Ecsit‐X4, which is highly expressed in the mitochondria of adult cardiomyocytes but down‐regulated in hypertrophic human heart samples and TAC‐treated mouse hearts. AAV9‐mediated Ecsit‐X4 gene therapy, administered either before or after TAC surgery, significantly attenuated cardiac hypertrophy. Cardiomyocyte‐specific Ecsit deficiency worsened TAC‐induced cardiac hypertrophy, while Ecsit‐X4 compensation independently rescued hypertrophic phenotypes in Ecsit^cKO^ mice. Mechanistically, ECSIT‐X4 localized to the mitochondria and interacted with STAT3, leading to increased STAT3 levels and enhanced serine 727 phosphorylation in cardiomyocyte mitochondria, thereby promoting strong mitochondrial bioenergetics. This study identified a novel transcript variant of ECSIT localized in the mitochondria of adult cardiomyocytes and highlights its potential as a therapeutic target for heart failure.

## Graphical abstract

ECSIT‐X4 is the predominant cardiac isoform of ECSIT, encoded by the Ecsit‐X4 transcript variant in adult cardiomyocytes. The splicing junction of exon 9 generates the mouse Ecsit‐X4 isoform, resulting in a recognizable C‐terminal sequence. It is shown that ECSIT‐X4 interacts with STAT3, enhancing the activity of complex I, thereby promoting mitochondrial OXPHOS function during pathological cardiac hypertrophy.

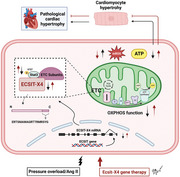

## 1 Introduction

Pressure overload caused by high blood pressure or valvular disease often leads to pathological cardiac hypertrophy, accompanied by maladaptive heart remodeling and cardiac dysfunction, which result in decreased compliance and an increased risk of heart failure.^[^ ^1^ ^]^ Nevertheless, the underlying molecular mechanisms of pathological cardiac hypertrophy are not fully established.

Mitochondrial dysfunction plays a vital role in the pathophysiology of the heart.^[^ ^2^ ^]^ The Energy supply in the form of ATP, generated through mitochondrial oxidative phosphorylation (OXPHOS), is essential for maintaining cardiac contraction and relaxation functions.^[^ ^3^ ^]^ Sustained cardiac overloading and activation of neurohumoral factors result in a decrease in the activity of myocardial mitochondrial respiratory chain complexes, leading to dysfunction in the mitochondrial OXPHOS system or uncoupling.^[^ ^4^ ^]^ Growing evidence indicates that diminished ATP production and excessive mitochondrial reactive oxygen species (mROS) are prominent during the decompensation stage of cardiac hypertrophy.^[^ ^5^ ^]^ Excessive mROS, as hazardous by‐products of energy metabolism emitted from the respiratory electron transport chain (ETC), can oxidize myocardial myofibrillar proteins, leading to a progressive decline in cardiac systolic function and irreversible damage to myocardial tissue structure.^[^ ^6^ ^]^ However, the regulatory molecules of myocardial mitochondrial function, especially during the development of pathological cardiac hypertrophy, remain to be elucidated.

ECSIT (evolutionary conserved signaling intermediate in Toll pathways) is a highly evolutionary conserved signaling molecule in the TLRs/IL‐1R signaling pathway.^[^ ^7^ ^]^ Previous studies have demonstrated that ECSIT interacts with the assembly chaperone NDUFAF1 to control the assembly or stability of mitochondrial complex I.^[^ ^8^ ^]^ Ecsit gene deletion leads to a profound alteration in macrophage energy metabolism, resulting in a striking shift to reliance on glycolysis and defective mitophagy.^[^ ^9^ ^]^ The C‐terminal domain of ECSIT was confirmed to directly bound to ACAD9, which coordinated the β‐oxidation of fatty acids and OXPHOS pathways to ensure efficient energy production.^[^ ^10^ ^]^ Furthermore, cardiac ECSIT has been described as a key factor in protecting mitochondria, helping to preserve normal cardiac structure and function.^[^ ^11^ ^]^ However, under the condition of pressure overload, whether and how ECSIT could ameliorate pathological cardiac hypertrophy and cardiac function deterioration as a result of cardiomyocyte mitochondrial dysfunction remains unclear.

The NCBI database showed five predicted transcript variants of mouse Ecsit (ID: 26940). A previous study comparing HEK293T mitochondrial lysates with total cell lysates revealed the additional presence of ≈50‐kDa ECSIT in the total cell lysates, confirming that the ≈45‐kDa ECSIT is specifically localized within mitochondrial complexes.^[^ ^8^ ^]^ Three cDNAs‐Ecsit1, Ecsit2, and Ecsit3‐were cloned by screening mouse pre‐B‐cell and liver cDNA libraries and are derived from a single gene through alternative splicing. In Ecsit1, exon 5 is utilized in a different reading frame compared to Ecsit2 and Ecsit3, as indicated by a black box in Ecsit1, contrasting with the open boxes in Ecsit2 and Ecsit3.^[^ ^12^ ^]^ Ten Ecsit splice isoforms were predicted by the EBI Alternative Splicing Database, of which only two isoforms have been annotated. These isoforms encode proteins with predicted molecular masses of ≈50‐ and 33‐kDa, respectively.^[^ ^8^ ^]^ Therefore, it remains to be determined whether spliced Ecsit is generated in cardiac cells, and if so, whether it is involved in mitochondrial bioenergetics and the development of pathological cardiac hypertrophy.

Previous studies have extensively explored the role of STAT3 in cardiac hypertrophy and heart failure through its nuclear transcription function.^[^ ^13^ ^]^ Besides its genomic functions, mitochondrial STAT3, also as a promising target, can control mitochondrial quality.^[^ ^13^ ^]^ There is evidence supporting a causal role for STAT3 activation in mediating cardio‐protection by maintaining mitochondrial function.^[^ ^13^ ^]^ STAT3 has been shown to translocate to the mitochondria, where it activates the electron transport chain via phosphorylation at serine 727 (S727).^[^ ^14^ ^]^ Increased mitochondrial STAT3 and its phosphorylation at S727 in the myocardium enhance mitochondrial complex activity, promote ATP synthesis, and inhibit mROS production.^[^ ^15^ ^]^ However, the mechanism by which STAT3 regulates mitochondrial function has not been fully clarified.

This study is the first to identify the previously unknown splice variant Ecsit‐X4 in adult cardiomyocyte mitochondria, and its down‐regulation in both TAC‐induced hypertrophic mouse hearts and human samples from individuals with hypertrophic heart conditions. We demonstrated the novel role of ECSIT‐X4 in mitigating pressure overload‐induced pathological cardiac hypertrophy by interacting with and regulating mitochondrial STAT3, thereby modulating OXPHOS function.

## 2 Results

### 2.1 ECSIT‐X4 is the Predominant Cardiac ECSIT Isoform and is Down‐Regulated in Adult Human and Murine Hypertrophic Hearts

**Figure 1:**
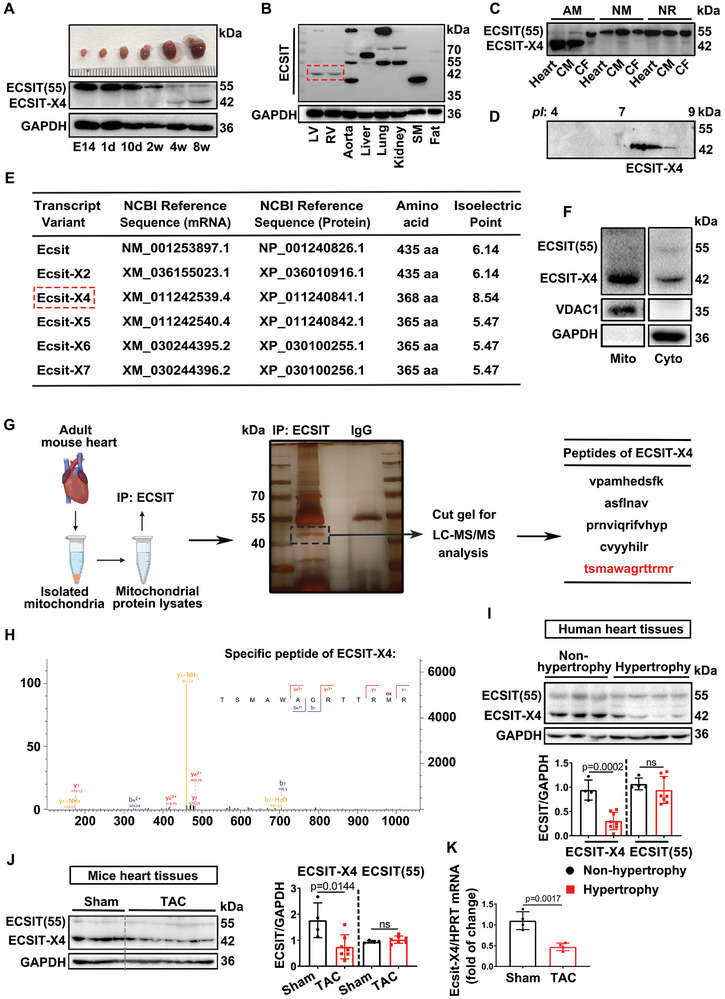
The ~42‐kDa ECSIT is encoded by transcript variant Ecsit‐X4 in adult cardiomyocytes and is down‐regulated in hypertrophic heart tissues. A) Representative Western blot of ECSIT protein expression and gross appearance of whole hearts in mouse hearts at different ages. *n* = 3 independent experiments. B) Representative Western blot of ECSIT protein in different organs of 8‐week‐old mice. Left ventricle (LV), Right ventricle (RV), Skeletal muscle (SM). *n* = 3 independent experiments. C) Representative Western blot showing ECSIT protein expression in heart tissues or cardiac cells derived from 8‐week‐old mice, 1‐ to 3‐day‐old neonatal mice, and 1‐ to 3‐day‐old neonatal rats. Adult mice (AM), Neonatal mice (NM), Neonatal rats (NR), Cardiomyocytes (CM), Cardiac fibroblasts (CF). *n* = 3 independent experiments. D) Molecular weight and *pI* of ECSIT were detected by 2‐DE. *n* = 1 independent experiment. E) Five predicted Ecsit transcript variants (Ecsit‐X2, Ecsit‐X4, Ecsit‐X5, Ecsit‐X6, Ecsit‐X7) from the NCBI database (ID: 26940) and the *pI* of each protein are shown in the table. The *pI* of each protein was obtained from https://web.expasy.org/protparam/. F) ECSIT protein levels in mitochondrial and cytoplasmic lysates extracted from the hearts of adult wild‐type mice (8 weeks old). *n* = 3 independent experiments. G) Mitochondrial protein lysates isolated from the hearts of 8‐week‐old adult mice were incubated with either anti‐ECSIT or anti‐IgG antibody, followed by electrophoresis and silver staining. The gel corresponding to the ≈42‐kDa band was then excised for LC‐MS/MS analysis, and five peptide sequences of ECSIT‐X4 were found in the immunocomplex. *n* = 1 independent experiment. H) LC‐MS scans of the specific peptide sequence “tsmawagrttrmr” matched Ecsit‐X4 encoded protein. *n* = 1 independent experiment. I) Representative Western blot and statistical results of ECSIT protein expression in non‐hypertrophic and hypertrophic human hearts. *n* = 4 or 8 samples per group. J) Male mice aged 8 weeks underwent either Sham or TAC surgery for 4 weeks. Representative Western blot and statistical results of ECSIT protein expression in Sham‐ or TAC‐treated mouse hearts were shown. *n* = 4 or 7 mice per group. K) The mRNA level of Ecsit‐X4 in Sham‐ or TAC‐treated mouse hearts. It was normalized to HPRT. *n* = 4 mice per group. Data were presented as mean ± SD, *p*‐values were determined by unpaired *t‐*test. *p <* 0.05 was considered statistically significant.

The protein level and molecular characteristics of ECSIT were detected in mouse hearts using a commercial ECSIT antibody (ab21288, Abcam). Intriguingly, a low molecular weight form of ECSIT (~42‐kDa) was found to be highly expressed in adult murine hearts as they reached maturity at 4 weeks of age (**Figure** **1**A). Notably, immunoblots showed that ~42‐kDa ECSIT is specifically highly expressed in the adult mouse hearts but not in the other organs (Figure 1B). Furthermore, we observed that the ~42‐kDa ECSIT isoform is predominantly expressed in cardiomyocytes of adult mice, while its expression is low in both adult and neonatal cardiac fibroblasts (CF) as well as neonatal cardiomyocytes (CM) (Figure 1C). Five predicted transcript variants of Ecsit (Ecsit‐X2, Ecsit‐X4, Ecsit‐X5, Ecsit‐X6, Ecsit‐X7) in mice are listed in the NCBI database (ID: 26940), encoding proteins with three distinct theoretical isoelectric points (*pI*) (Figure 1E). 2D gel electrophoresis (2‐DE) was conducted to determine the *pI* and relative molecular weights of ECSIT proteins. Figure 1D shows a prominent dot near 42‐kDa, with a *pI* close to 8, consistent with the protein encoded by Ecsit‐X4. It has been reported that ECSIT can localize to mitochondria.^[^ ^8^ ^]^ Accordingly, mitochondrial and cytoplasmic proteins were extracted from adult mouse hearts, and Western blot analysis revealed that ECSIT‐X4 was predominantly expressed in mitochondria, with low expression in the cytoplasm under physiological conditions (Figure 1F). To further confirm whether the ~42‐kDa ECSIT is encoded by the Ecsit‐X4 splice variant, mitochondrial proteins were isolated from adult mouse hearts. Immunoprecipitation was performed using an anti‐ECSIT antibody, and the gel at the 42‐kDa position was excised, followed by liquid chromatography‐tandem mass spectrometry (LC‐MS/MS) analysis. Five peptide sequences of ECSIT‐X4 were identified in the immunocomplex (Figure 1G). Notably, the unique peptide sequence “tsmawagrttrmr” matched exclusively to the Ecsit‐X4 encoding protein (Figure 1H), indicating that the ~42‐kDa ECSIT is the predominant isoform expressed in the mitochondria of adult mouse hearts. Additionally, we confirmed that the splice junction of exon 9 in murine Ecsit‐X4 is responsible for encoding an ECSIT protein with a unique C‐terminal sequence, which accounts for its lower molecular weight (~42‐kDa). To investigate the role of ECSIT‐X4 in pathological cardiac hypertrophy, we first examined changes in ECSIT‐X4 expression in myocardial samples from non‐hypertrophic donors and patients undergoing cardiac valve surgery (Figure S1B, Supporting Information). We observed that ECSIT‐X4 was notably down‐regulated in hypertrophic human hearts (Figure 1I), and Masson's trichrome staining showed exaggerated collagen deposition in hypertrophic heart sections compared to those from donors (Figure S1A, Supporting Information). Additionally, both the protein and mRNA levels of ECSIT‐X4 were decreased in the hearts of TAC‐treated mice (Figure 1J,K). In conclusion, ECSIT‐X4 is the predominant isoform in adult human and murine hearts and is down‐regulated in pathological cardiac hypertrophy.

### 2.2 Ecsit‐X4 Gene Delivery Before or After TAC Alleviates Pathological Cardiac Hypertrophy

**Figure 2:**
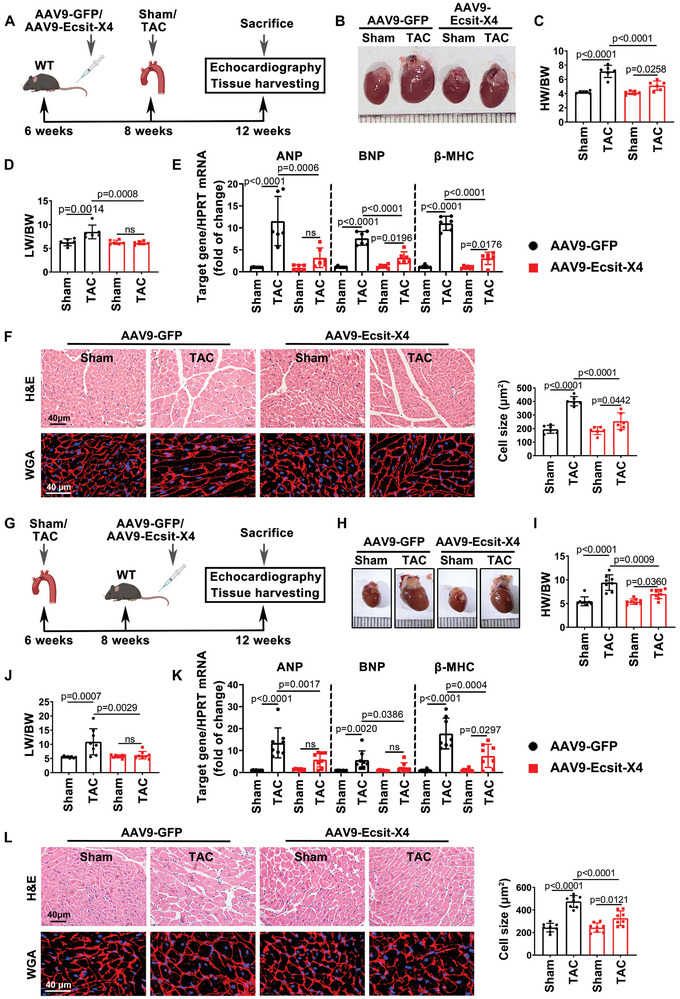
Ecsit‐X4 gene delivery before or after TAC attenuates pressure overload‐induced pathological cardiac hypertrophy. A) Treatment regimen. B) Representative gross appearance of whole hearts. Scale bar = 1 mm. C,D) The ratios of HW/BW and LW/BW were calculated. heart weight (HW), body weight (BW), lung weight (LW). E) The mRNA levels of ANP, BNP, and β‐MHC. All were normalized to HPRT. Atrial natriuretic peptide (ANP), Brain natriuretic peptide (BNP), and β‐myosin heavy chain (β‐MHC). F) Hematoxylin and eosin (H&E) and Wheat germ agglutinin (WGA) staining were performed to detect cardiomyocyte cross‐sectional area. Statistical result of cell size was presented. Scale bar = 40 µm. *n* = 6 mice per group. G) Treatment regimen. H) Representative gross appearance of whole hearts. Scale bar = 1 mm. I,J) The ratios of HW/BW and LW/BW were calculated. K) The mRNA levels of ANP, BNP, and β‐MHC. All were normalized to HPRT. L) Hematoxylin and eosin (H&E) and Wheat germ agglutinin (WGA) staining were performed to detect cardiomyocyte cross‐sectional area. Statistical result of cell size was presented. Scale bar = 40 µm. *n* = 8 mice per group. Data were presented as mean ± SD, *p*‐values were determined by one‐way ANOVA corrected by the post hoc Turkey's test. *p <* 0.05 was considered statistically significant.

Given the well‐known function of ECSIT‐X4 as a therapeutic target for cardiac hypertrophy, mice were subjected to TAC or sham surgery after or before being randomly assigned to receive AAV9‐Ecsit‐X4 or control administration (**Figure** **2**A,G; Figures S2A,B and S3A, Supporting Information). Figure 2B–D reveals that overexpression of Ecsit‐X4 before TAC surgery contributed to a smaller heart size and lower ratios of heart weight (HW)/body weight (BW), and lung weight (LW)/ body weight (BW). In AAV9‐Ecsit‐X4 pre‐treated TAC‐mice group, the mRNA levels of hypertrophy‐related genes, including atrial natriuretic peptide (ANP), brain natriuretic peptide (BNP), and β‐myosin heavy chain (β‐MHC), were significantly down‐regulated (Figure 2E). Consistent with these findings, morphological analysis revealed that ECSIT‐X4 pre‐treatment reduced the cross‐sectional area of cardiomyocytes in TAC‐mice (Figure 2F). Additionally, excessive cardiac collagen synthesis and deposition were also suppressed in the hearts of AAV9‐Ecsit‐X4 pre‐treated TAC‐mice (Figure S2C–E, Supporting Information). Echocardiographic analysis showed that TAC significantly increased left ventricular posterior wall thickness (LVPW), interventricular septum thickness (IVS), and left ventricular internal dimension (LVID) in AAV9‐GFP pre‐treated mice. However, these parameters were markedly reduced in AAV9‐Ecsit‐X4 pre‐treated TAC‐mice (Figure S2F–K, Supporting Information). Moreover, while TAC significantly decreased ejection fraction (EF%) and fractional shortening (FS%) in control virus‐transfected mice, marked improvements were observed in AAV9‐Ecsit‐X4 pre‐treated TAC‐mice (Figure S2L,M, Supporting Information). Gene therapy data further demonstrated that overexpression of Ecsit‐X4 after TAC surgery alleviated cardiac hypertrophy, fibrosis, and cardiac dysfunction (Figure 2G–L; Figure S3B–L, Supporting Information). Overall, these results indicate that ECSIT‐X4 is crucial not only for the prevention but also for the treatment of established cardiac hypertrophy.

### 2.3 Compensation of Ecsit‐X4 Rescues the Consequences of Pressure Overload‐Induced Cardiac Hypertrophy in Ecsit^cKO^ Mice

**Figure 3:**
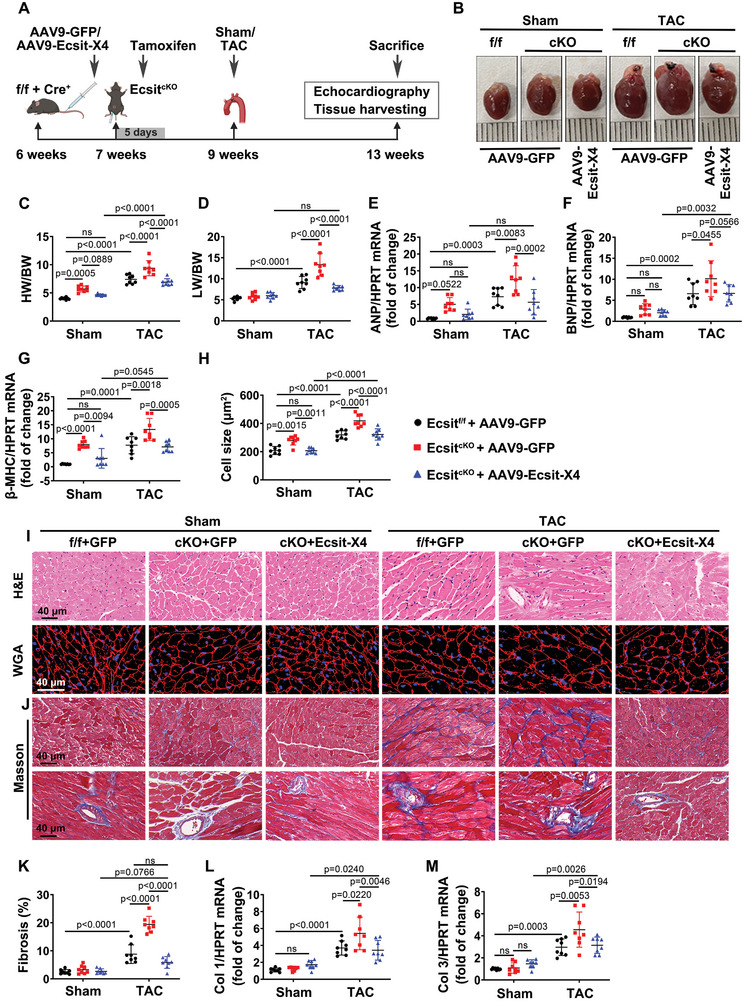
Compensation of Ecsit‐X4 alleviates TAC‐induced pathological cardiac hypertrophy in Ecsit^cKO^ mice. A) Treatment regimen. B) Representative gross appearance of whole hearts. Scale bar = 1 mm. C,D) The ratios of HW/BW and LW/BW. E–G) The mRNA expression of ANP, BNP, and β‐MHC. All were normalized to HPRT. H‐K) H&E, WGA staining, and Masson's trichrome staining were performed to detect cardiomyocyte cross‐sectional area and myocardial collagen in mouse hearts. Scale bar = 40 µm. Statistical results of cell size and fibrosis were shown in (H,K), respectively. L,M) The mRNA levels of Collagen I (Col 1) and Collagen III (Col 3). All were normalized to HPRT. *n* = 8 mice per group. Data were presented as mean ± SD, *p*‐values were determined by two‐way ANOVA corrected by the post hoc Turkey's test. *p <* 0.05 was considered statistically significant.

Next, we sought to determine the independent functional involvement of ECSIT‐X4 in pressure overload‐induced cardiac hypertrophy. Ecsit^f/f^ mice were crossed with Myh6‐Cre^ERT^ mice, and AAV9‐Ecsit‐X4 was administered to them 1 week before treatment with 50 mg kg^−1^ day^−1^ of tamoxifen for 5 days to conditionally knock out Ecsit in the cardiomyocytes. After 9 days, the mice underwent TAC surgery for 4 weeks (**Figure** **3**A; Figure S4A,B, Supporting Information). Cardiac size and mass were significantly increased in Ecsit^cKO^ mice at baseline, accompanied by a moderate decline in cardiac function. However, no significant phenotypic differences were observed between AAV9‐Ecsit‐X4‐transfected Ecsit^cKO^ mice and AAV9‐GFP‐transfected Ecsit^f/f^ mice, all of which underwent Sham surgery (Figure 3B,C; Figure S4C–J, Supporting Information). Additionally, TAC surgery induced a larger heart size, higher ratios of HW/ BW, and LW/BW, as well as higher mRNA levels of hypertrophic‐related genes, including ANP, BNP and β‐MHC in Ecsit^cKO^ mice. Nevertheless, these parameters were substantially suppressed by AAV9‐mediated compensation of Ecsit‐X4 (Figure 3B–G). Consistently, morphological analysis revealed that the enlarged cross‐sectional area of cardiac myocytes, along with increased interstitial and perivascular fibrosis, was further aggravated in TAC‐induced Ecsit^cKO^ mouse hearts but was reversed by the compensation of Ecsit‐X4 (Figure 3H–K). Additionally, the increased mRNA expression of Collagen I and Collagen III was reduced in TAC‐induced Ecsit^cKO^ mouse hearts following AAV9‐mediated compensation of Ecsit‐X4 (Figure 3L,M). Echocardiographic analysis showed that cardiomyocyte‐specific Ecsit gene knockout aggravated TAC‐induced increase in LVPW, IVS, and LVID. However, compensation of Ecsit‐X4 reversed these parameters (Figure S4C–H, Supporting Information). Additionally, Ecsit^cKO^ mice subjected to TAC surgery also exhibited a further decrease in EF% and FS%, but an apparent reversion was observed in AAV9‐Ecsit‐X4 injection group (Figure S4I,J, Supporting Information). These data demonstrate that compensation of Ecsit‐X4 alleviates the adverse consequences of pressure overload‐induced cardiac hypertrophy in Ecsit^cKO^ mice.

### 2.4 Overexpression of Ecsit‐X4 Inhibited Ang II‐Induced Cardiomyocyte Hypertrophy In Vitro

**Figure 4:**
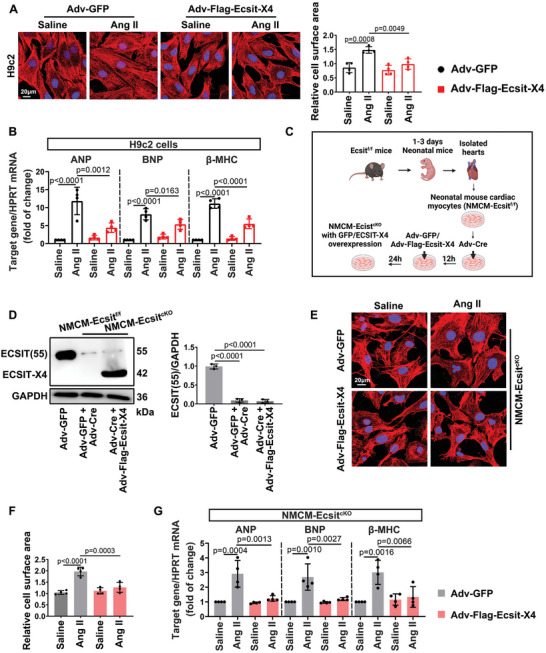
Overexpression of Ecsit‐X4 protects cardiomyocytes from Ang II‐induced hypertrophy. A) H9c2 cells transfected with Adv‐GFP or Adv‐Flag‐Ecsit‐X4 for 24 h before they were treated with Saline or Ang II (1 µm) for 48 h. Cardiomyocyte surface areas were analyzed by immunofluorescence staining with anti‐α‐actinin antibody (red) and DAPI (blue). Statistical result of cell surface areas was shown. Scale bar = 20 µm. *n* = 4 independent experiments. B) The mRNA levels of ANP, BNP, and β‐MHC in H9c2 cells. All were normalized to HPRT. *n* = 4 independent experiments. C) Neonatal mouse primary cardiomyocytes (NMCMs) isolated from Ecsit^f/f^ mice were transfected with Adv‐GFP or Adv‐Cre for 12 h to delete the Ecsit gene (NMCM‐Ecsit^cKO^), and then transfected with Adv‐GFP or Adv‐Flag‐Ecsit‐X4 for another 24 h. D) Representative Western blot and statistical result of ECSIT‐X4 and ~55‐kDa ECSIT were shown. *n* = 3 independent experiments. E, F) NMCM‐Ecsit^cKO^ were transfected with Adv‐GFP or Adv‐Flag‐Ecsit‐X4 for 24 h followed by Saline or Ang II stimulation for 48 h. Cardiomyocyte surface areas of NMCM‐Ecsit^cKO^ stained with α‐actinin (red) and DAPI (blue) were shown. Statistical result of cell surface areas was shown. Scale bar = 20 µm. *n* = 4 independent experiments. G) The mRNA levels of ANP, BNP, and β‐MHC in NMCM‐Ecsit^cKO^. All were normalized to HPRT. *n* = 4 independent experiments. Data were presented as mean ± SD, *p*‐values were determined by one‐way ANOVA corrected by the post hoc Turkey's test. *p <* 0.05 was considered statistically significant.

To determine whether ECSIT‐X4 regulates hypertrophic growth of cardiomyocytes, we assessed the effect of manipulating Ecsit‐X4 expression on Ang II‐induced cardiomyocyte hypertrophy. Ang II‐induced enlargement of H9c2 cells was markedly suppressed by transfection with Adv‐Flag‐Ecsit‐X4, leading to reduced cell surface area and attenuated expression of hypertrophy‐related genes (**Figure** **4**A,B). To further verify that ECSIT‐X4 regulates cardiomyocyte hypertrophy independently of other ECSIT isoforms, neonatal mouse cardiomyocytes (NMCMs) isolated from Ecsit^f/f^ mice (NMCM‐Ecsit^f/f^) were transfected with Adv‐Cre to delete the Ecsit gene (NMCM‐Ecsit^cKO^), followed by transfection with either Adv‐GFP or Adv‐Flag‐Ecsit‐X4 (Figure 4C,D). As predicted, NMCM‐Ecsit^cKO^ overexpressing Ecsit‐X4 exhibited alleviative hypertrophy, as evidenced by a smaller surface area and decreased mRNA levels of hypertrophy‐related genes (Figure 4E–G). These data indicated that ECSIT‐X4 plays an independent role in protecting against hypertrophic growth of cardiomyocytes.

### 2.5 Overexpression of Ecsit‐X4 Enhances Mitochondrial OXPHOS Function in TAC‐Treated Mouse Hearts and Ang II‐Treated Cardiomyocytes

**Figure 5:**
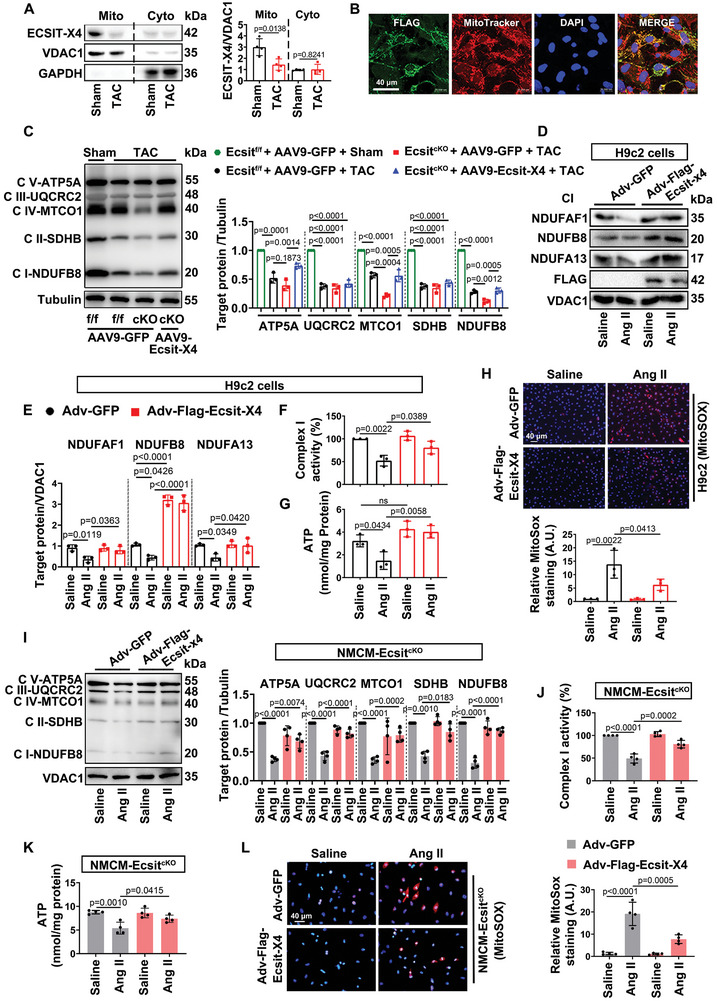
Overexpression of Ecsit‐X4 enhances mitochondrial OXPHOS function in TAC‐induced hypertrophic mouse hearts and Ang II‐induced hypertrophic cardiomyocytes. A) Representative Western blot and statistical result of ECSIT‐X4 in myocardial mitochondria isolated from Sham‐ or TAC‐treated mouse hearts. *n* = 4 mice per group. B) NMCMs were transfected with Adv‐Flag‐Ecsit‐X4 for 24 h. Immunofluorescence staining with anti‐Flag antibody (green), mitochondria marker Mitotracker (red), and DAPI (blue). Scale bar = 40 µm. *n* = 3 independent experiments. C) Representative Western blot and statistical results of the ETC complex components in mouse hearts. *n* = 3 mice per group. D,E) Representative Western blots and statistical results of NDUFAF1, NDUFB8, and NDUFA13 in mitochondria of H9c2 cells. *n* = 3 independent experiments. F) The activity of mitochondrial complex I in H9c2 cells. *n* = 3 independent experiments. G) ATP content in H9c2 cells. *n* = 3 independent experiments. H) H9c2 cells were incubated with MitoSox. Fluorescence measurement was performed to detect mROS, and statistical result of relative MitoSox staining was shown. Scale bar = 40 µm. *n* = 3 independent experiments. I) Representative Western blot and statistical results of the ETC complex components in NMCM‐Ecsit^cKO^. *n* = 4 independent experiments. J) The activity of mitochondrial complex I in NMCM‐Ecsit^cKO^. *n* = 4 independent experiments. K) ATP content in NMCM‐Ecsit^cKO^. *n* = 4 independent experiments. L) NMCM‐Ecsit^cKO^ were incubated with MitoSox. Fluorescence measurement was performed to detect mROS, and statistical result of relative MitoSox staining was shown. Scale bar = 40 µm. *n* = 4 independent experiments. Data were presented as mean ± SD, *p*‐values were determined by unpaired *t‐*test and one‐way ANOVA corrected by the post hoc Turkey's test. *p <* 0.05 was considered statistically significant.

The inability of mitochondria to adequately supply ATP, leading to energy depletion, is central to the loss of contractile function in pathological cardiac hypertrophy and heart failure.^[^ ^16^ ^]^ It is well established that ECSIT copurifies with OXPHOS assembly chaperones.^[^ ^9^ ^]^ Our data show that ECSIT‐X4 is predominantly localized in the mitochondria of adult hearts (Figure 1F). Building on this, we next investigated alterations in ECSIT expression within the mitochondria of mouse hearts under TAC‐induced cardiac hypertrophy. As shown in **Figure** **5**A, the predominant immunodetection of ECSIT‐X4 in the mitochondria was reduced in hypertrophic mouse hearts. We then investigated whether exogenous FLAG‐ECSIT‐X4 could localize to mitochondria in cardiomyocytes. Immunofluorescence analysis confirmed that FLAG‐tagged ECSIT‐X4 was present in the mitochondria of NMCMs (Figure 5B). To evaluate whether ECSIT‐X4 could enhance mitochondrial bioenergetics in vivo, we assessed the expression of mitochondrial ETC complex subunits. Western blot analysis revealed that Ecsit deficiency in cardiomyocytes significantly down‐regulated ETC complex components following TAC surgery, compared to Ecsit^f/f^ mice. Notably, the reintroduction of ECSIT‐X4 independently restored the levels of these proteins (Figure 5C). Furthermore, to confirm that ECSIT‐X4 regulates mitochondrial function in hypertrophic cardiomyocytes, we evaluated the expression levels of key complex subunits in vitro. Ang II‐stimulated H9c2 cells exhibited reduced levels of mitochondrial complex assembly chaperones. However, mitochondria isolated from H9c2 cells transfected with Adv‐Flag‐Ecsit‐X4 showed increased expression of these complex subunits (Figure 5D,E; Figure S5A,B, Supporting Information). Consistent with these findings, Ecsit‐X4 overexpression enhanced complex I activity and increased ATP content in Ang II‐treated H9c2 cells (Figure 5F,G). Conversely, high protein level of ECSIT‐X4 suppressed the Ang II‐induced increase in mitochondria‐derived ROS (mROS) (Figure 5H). Figure 5I–L shows that, upon deletion of the endogenous Ecsit gene, overexpression of Ecsit‐X4 can independently protect cardiomyocytes from Ang II‐induced mitochondrial dysfunction. This is evidenced by increases in ETC complex levels, complex I activity, and ATP production, as well as a reduction in mROS. These results indicate that Ecsit‐X4 overexpression can alleviate Ang II‐induced mitochondrial OXPHOS dysfunction, both in the presence of all ECSIT isoforms and independently by providing a protective effect in the absence of the Ecsit gene in hypertrophic cardiomyocytes.

### 2.6 ECSIT‐X4 Interacts with STAT3 and Increases its Serine 727 Phosphorylation in the Mitochondria of Hypertrophic Cardiomyocytes

**Figure 6:**
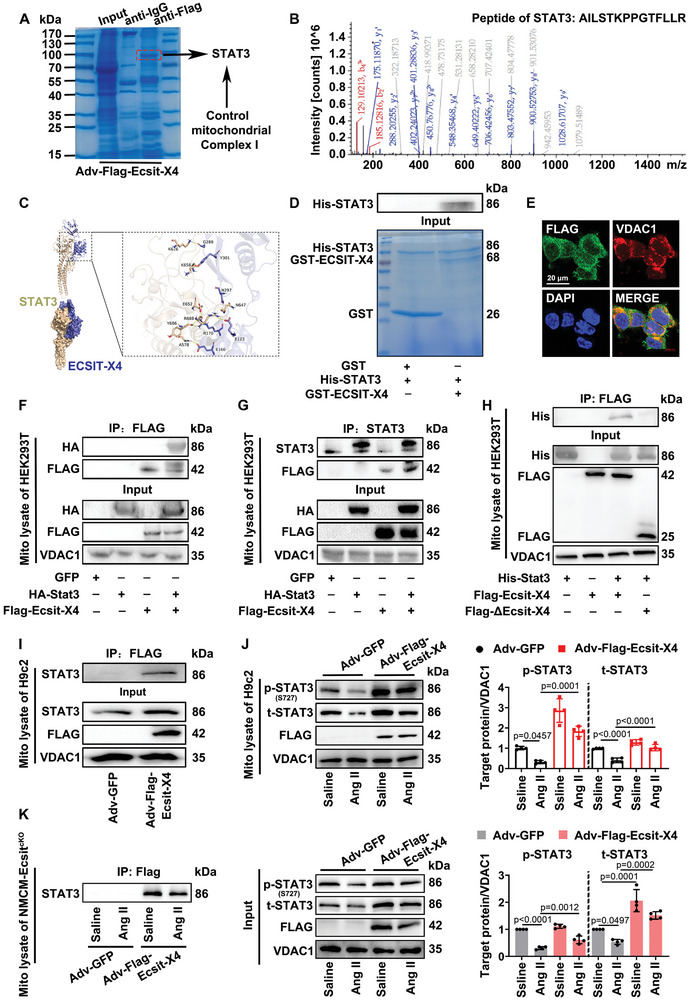
ECSIT‐X4 interacts with STAT3 and enhanced its serine 727 phosphorylation in the mitochondria of hypertrophic cardiomyocytes. A) NMCMs were isolated from Ecsit^f/f^ mice transfected with Adv‐Flag‐Ecsit‐X4. Mitochondria isolated from the NMCMs were lysed and immunoprecipitated with either anti‐IgG or anti‐Flag antibody. Flag‐labeled immunoprecipitates were visualized using Coomassie blue staining and subsequently subjected to LC‐MS/MS analysis to identify the target protein (STAT3), a mitochondrial complex I‐related protein that interacts with ECSIT‐X4. B) LC‐MS/MS fragment analysis of the specific peptide segment (AILSTKPPGTFLLR) of the STAT3 protein. The X‐axis represents the mass‐to‐charge ratio (m/z), while the Y‐axis represents signal intensity. Red line: b ions; blue lines: y ions; gray line: fragment ions. *n* = 1 independent experiments. C) The structure of ECSIT‐X4 protein was build using AlphaFold2, and the interaction between ECSIT‐X4 (blue) and STAT3 (yellow) was predicted by ZDOCK (version 3.0.2). Score = 1408.166. D) Interaction between ECSIT‐X4 and STAT3 was demonstrated by GST‐pull down. *n* = 3 independent experiments. E) HEK293T cells were transfected with expression plasmid encoding Flag‐ECSIT‐X4 for 24 h. Immunofluorescence staining with anti‐Flag antibody (green), mitochondria marker VDAC1 (red), and DAPI (blue). Scale bar = 20 µm. *n* = 3 independent experiments. F,G) HEK293T cells were co‐transduced with expression plasmid encoding FLAG‐ECSIT‐X4 and HA‐STAT3 for 36 h, and interaction between ECSIT‐X4 and STAT3 in mitochondria was determined by immunoprecipitation. *n* = 3 independent experiments. H) HEK293T cells were co‐transduced with expression plasmid encoding His‐STAT3 and FLAG‐ECSIT‐X4 or FLAG‐ΔECSIT‐X4 (mutant with 110–310 amino acid) for 36 h, and interaction between STAT3 and ECSIT‐X4 or ΔECSIT‐X4 in mitochondria was determined by immunoprecipitation. *n* = 3 independent experiments. I) Interaction between FLAG‐ECSIT‐X4 and endogenous STAT3 in mitochondria of H9c2 cells was determined by immunoprecipitation with anti‐Flag antibody followed by immunoblot with anti‐STAT3 antibody. *n* = 3 independent experiments. J) Representative Western blots and statistical results of total STAT3 and p (S727)‐STAT3 were shown. *n* = 3 independent experiments. K) Interaction between FLAG‐ECSIT‐X4 and endogenous STAT3 in the mitochondria of NMCM‐Ecsit^cKO^ is illustrated, along with representative Western blots and statistical results for total STAT3 and p (S727)‐STAT3. *n* = 4 independent experiments. Data were presented as mean ± SD, *p*‐values were determined by one‐way ANOVA corrected by the post hoc Turkey's test. *p <* 0.05 was considered statistically significant.

Next, we investigated the underlying molecular mechanism involved in ECSIT‐X4‐mediated enhancement of mitochondrial function in hypertrophic cardiomyocytes. NMCMs isolated from Ecsit^f/f^ mice were transfected with Adv‐Flag‐Ecsit‐X4. Mitochondria isolated from NMCMs were lysed and immunoprecipitated using either anti‐IgG or anti‐Flag antibody. Coomassie blue staining revealed that the Flag immunoprecipitates contained significantly more protein compared to the IgG immunoprecipitates (**Figure** **6**A). Furthermore, LC‐MS/MS analysis of the Flag immunoprecipitates identified STAT3 as a potential target of Flag‐ECSIT‐X4, which is associated with mitochondrial complex I activity (Figure 6A,B). The protein structure of ECSIT‐X4 was determined using AlphaFold2, and its interaction with the mitochondria‐related protein STAT3 was predicted by ZDOCK (Figure 6C), suggesting the possibility of an interaction between ECSIT‐X4 and STAT3. To confirm this interaction, we conducted a GST pull‐down assay, which revealed that His‐tagged STAT3 was present in the GST‐tagged ECSIT‐X4 pull‐down complex (Figure 6D). To further confirm the interaction between ECSIT‐X4 and STAT3 in mitochondria, HEK293T cells were co‐transfected with expression plasmids encoding FLAG‐ECSIT‐X4 and HA‐STAT3. Immunofluorescence analysis showed that Flag‐ECSIT‐X4 was localized in the mitochondria of HEK293T cells (Figure 6E). Consistently, a co‐immunoprecipitation assay demonstrated that ECSIT‐X4 interacted with STAT3 in mitochondria isolated from HEK293T cells (Figure 6F,G). Additionally, a mutant of the 111–310 amino acid segment of ECSIT‐X4 (ΔECSIT‐X4) was constructed based on molecular docking data. Co‐immunoprecipitation assays revealed that ΔECSIT‐X4 did not bind to mitochondrial STAT3 in HEK293T cells (Figure 6H). STAT3 localized in the mitochondria supports mitochondrial function, and its phosphorylation at S727 is required.^[^ ^17^ ^]^ Next, ECSIT‐X4 was overexpressed in cultured cardiomyocytes to determine whether it can bind to mitochondrial STAT3 and affect the phosphorylation of STAT3 at S727 under Ang II‐induced pathological conditions. Figure 6I,J shows that ECSIT‐X4 interacted with mitochondrial STAT3, and its overexpression reversed the down‐regulation of total and S727 phosphorylation levels induced by Ang II in cultured H9c2 cells. Furthermore, we also used NMCM‐Ecsit^cKO^ to explore whether the regulatory effect of ECSIT‐X4 on STAT3 is influenced by other ECSIT isoforms. Figure 6K shows that overexpression of ECSIT‐X4 significantly increased STAT3 levels, both total and phosphorylated at S727, in the mitochondria of NMCM‐Ecsit^cKO^ cells after Ang II stimulation. In conclusion, these results demonstrate that ECSIT‐X4 forms a complex with STAT3 in the mitochondria, resulting in the up‐regulation of STAT3 protein and S727 phosphorylation level in hypertrophic cardiomyocytes. Importantly, this regulatory effect is not influenced by other ECSIT isoforms.

### 2.7 ECSIT‐X4 Regulates Mitochondrial OXPHOS Function in Hypertrophic Cardiomyocytes by Activating Mitochondrial STAT3

**Figure 7:**
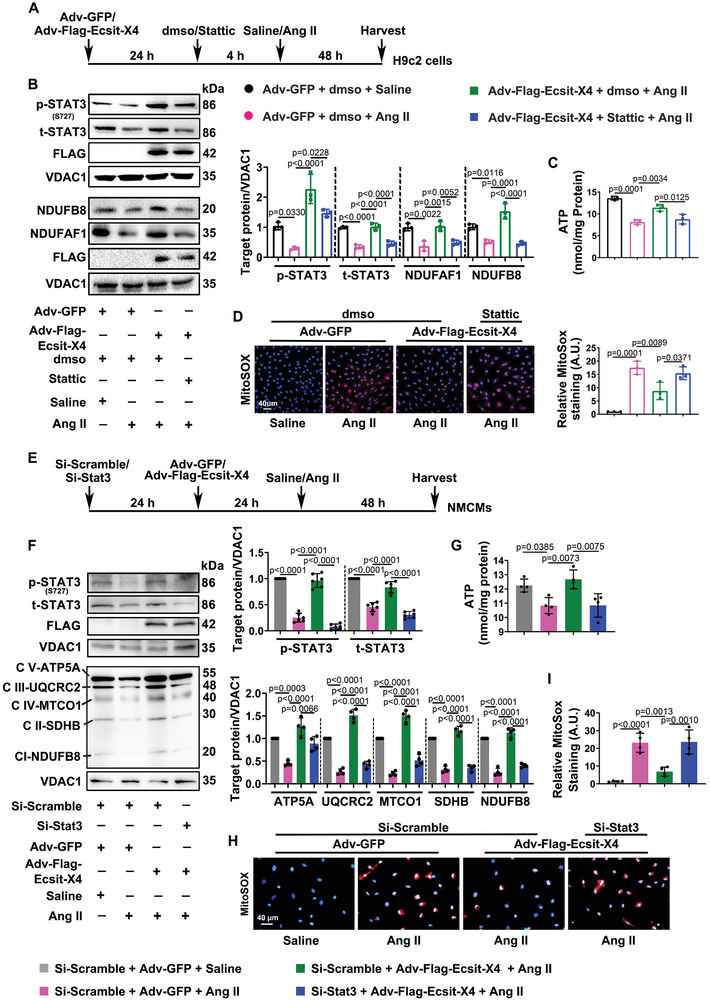
Overexpression of Ecsit‐X4 enhances mitochondrial OXPHOS function via STAT3 in hypertrophic cardiomyocytes. A) H9c2 cells were transfected with Adv‐Flag‐Ecsit‐X4 or Adv‐GFP for 24 h, and then were treated with 5 µm Stattic or dmso for 4 h before Saline or Ang II stimulation for 48 h. B) Representative Western blots and statistical results of p (S727)‐STAT3, t‐STAT3, NDUFAF1 and NDUFB8 in mitochondria were presented. *n* = 3 independent experiments. C) ATP content in H9c2 cells. *n* = 3 independent experiments. D) H9c2 cells incubated with MitoSOX. Fluorescence measurement was performed to detect mROS, and statistical result of relative MitoSOX staining was shown. Scale bar = 40 µm. *n* = 3 independent experiments. E) NMCMs were transfected with Si‐Scramble or Si‐Stat3 for 24 h, and then were transfected with Adv‐Flag‐Ecsit‐X4 or Adv‐GFP for 24 h before Saline or Ang II stimulation for 48 h. F) Representative Western blots and statistical results of p (S727)‐STAT3, t‐STAT3 and ETC complex components in mitochondria were presented. *n* = 4 independent experiments. G) ATP content in NMCMs. *n* = 4 independent experiments. H,I) NMCMs incubated with MitoSox, and mROS was detected by fluorescence measurement. Statistical result of relative MitoSox staining was shown. Scale bar = 40 µm. *n* = 4 independent experiments. Data were presented as mean ± SD, *p*‐values were determined by one‐way ANOVA corrected by the post hoc Turkey's test. *p <* 0.05 was considered statistically significant.

To verify the role of ECSIT‐X4 in mitochondrial OXPHOS function through STAT3, we used Stattic to inhibit STAT3 activation (**Figure** **7**A). Western blots showed that overexpression of ECSIT‐X4 up‐regulated S727 phosphorylation and total protein level of STAT3, while Stattic administration abolished these changes in Ang II‐treated H9c2 cells (Figure 7B). Additionally, overexpression of ECSIT‐X4 increased the protein expression of key complex subunits and ATP content in Ang II‐stimulated H9c2 cells, but Stattic administration also negated these protective effects (Figure 7B,C). Examination of mROS level revealed that overexpression of ECSIT‐X4 could not prevent the increase in mROS content when Stattic was added to Ang II‐treated H9c2 cells (Figure 7D). These data give evidence that ECSIT‐X4 regulates the mitochondrial OXPHOS function in hypertrophic cardiomyocytes by activating mitochondrial STAT3. Then, we further validated whether suppressing the expression of STAT3 worsen mitochondrial OXPHOS function through siRNA‐mediated STAT3 knockdown (Figure 7E). Figure 7F,G demonstrates that knockdown of STAT3 significantly reversed the up‐regulation of mitochondrial complex levels and ATP content induced by ECSIT‐X4 overexpression in Ang II‐treated NMCMs. The level of mROS indicated that the protective effect of ECSIT‐X4 overexpression was significantly weakened by the suppression of STAT3 expression in Ang II‐treated NMCMs (Figure 7H,I). Collectively, these data suggest that ECSIT‐X4 contributes to the protection against cardiomyocyte hypertrophy by regulating mitochondrial OXPHOS function, at least in part, through its binding to STAT3, which enhances both its total protein level and S727 phosphorylation activity in the mitochondria.

### 2.8 STAT3 is Essential for the Regulatory Role of ECSIT‐X4 in Mitochondrial OXPHOS Function During Pressure Overload‐Induced Cardiac Hypertrophy

**Figure 8:**
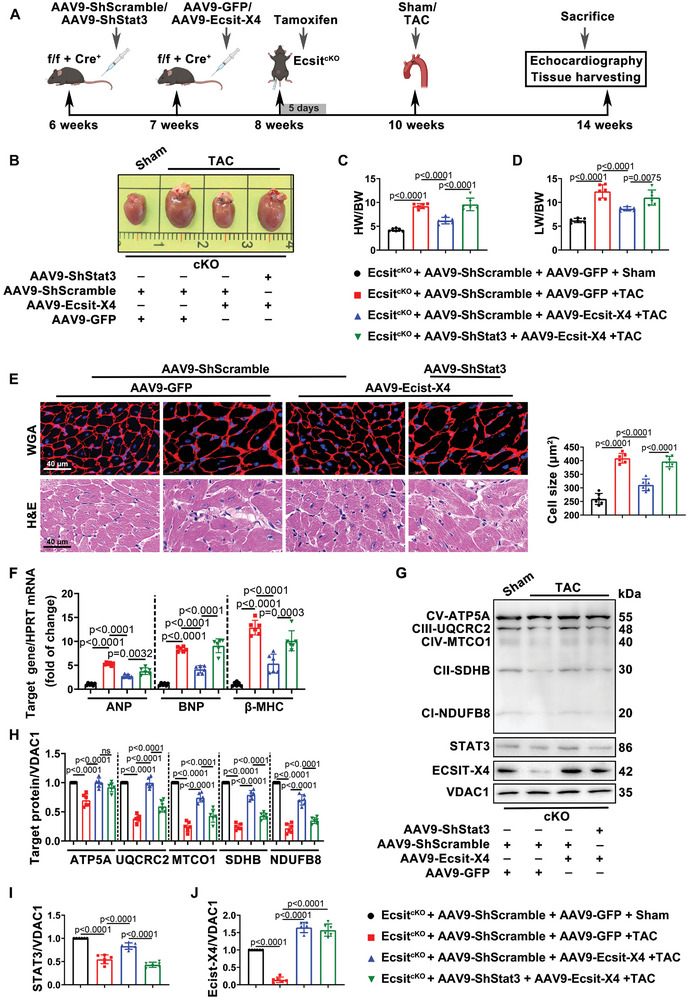
ECSIT‐X4 alleviates cardiac hypertrophy and enhances mitochondrial OXPHOS function through STAT3 in hypertrophic Ecsit^cKO^ mice. A) Treatment regimen. B) Representative gross appearance of whole hearts. Scale bar = 1 mm. C,D) The ratios of HW/BW and LW/BW. E) WGA and H&E staining were performed to detect cardiomyocyte cross‐sectional area in mouse hearts. Scale bar = 40µm. Statistical result of cell size was shown. F) The mRNA expression of ANP, BNP, and β‐MHC. All were normalized to HPRT. G–J) Representative Western blots and statistical results of STAT3, ECSIT‐X4, and ETC complex components in mitochondria were presented. *n* = 6 mice per group. Data were presented as mean ± SD, *p*‐values were determined by one‐way ANOVA corrected by the post hoc Turkey's test. *p <* 0.05 was considered statistically significant.

We engineered AAV9 viral vectors controlled by the cardiomyocyte‐specific cTnT promoter to knock down Stat3 in cardiomyocytes and determined its role in improving cardiac hypertrophy mediated by ECSIT‐X4 in vivo. Ecsit^f/f^ mice crossed with Myh6‐Cre^ERT^ mice were injected with AAV9‐ShStat3 for 1 week before receiving AAV9‐Ecsit‐X4, followed by tamoxifen administration via intraperitoneal injection for five consecutive days 1 week later. After a 9‐day rest period, TAC surgery was performed to induce cardiac hypertrophy over a 4‐week period (**Figure** **8**A). As predicted, transfection with AAV9‐Ecsit‐X4 significantly decreased cardiac size, mass, cross‐sectional area of cardiac myocytes, interstitial and perivascular fibrosis, and cardiac dysfunction in TAC‐treated Ecsit^cKO^ mice, but knockdown of Stat3 reversed these changes (Figure 8B–F; Figure S6A–K, Supporting Information). Reintroduction of Ecsit‐X4 in Ecsit^cKO^ mice restored the down‐regulated ETC complex components following TAC surgery, but suppressing the expression of STAT3 caused overexpression of ECSIT‐X4 to fail to protect the mitochondrial OXPHOS function (Figure 8G–J). Our data suggested that STAT3 is vital for the regulatory function of ECSIT‐X4 on pressure overload‐induced cardiac hypertrophy.

## 3 Discussion

Cardiac hypertrophy is the growth response to pathological stimuli that involves a complex biological process.^[^ ^18^ ^]^ In this study, we identified the previously unrecognized transcript variant Ecsit‐X4 as a key player in protection against mitochondrial dysfunction during pressure overload‐induced pathological cardiac hypertrophy. Additionally, we uncovered a novel mechanism by which ECSIT‐X4 regulates mitochondrial OXPHOS function through its interaction with STAT3, influencing mitochondrial bioenergetics.

The splicing of pre‐mRNA during the occurrence and development of pathological cardiac hypertrophy allows the generation of multiple splice isoforms from a given gene.^[^ ^19^ ^]^ New splice variants play a pivotal role in regulating gene expression, influencing the differentiation and specialization of cardiac cells, and offering novel biomarkers or potential therapeutic targets.^[^ ^20^ ^]^ Previous studies have shown that the Ecsit gene is transcribed and spliced into mRNAs of varying lengths, resulting in multiple ECSIT proteins of different molecular weights.^[^ ^8^, ^12^ ^]^ A seminal study demonstrated that the ≈45‐kDa ECSIT, which copurifies with the OXPHOS assembly chaperone NDUFAF1, is localized within the mitochondrial outer and inner membranes in HEK293T cells, while the ≈50‐kDa ECSIT is found in the cytoplasm and nucleus.^[^ ^8^ ^]^ In this study, we demonstrated that the previously underappreciated ≈42‐kDa ECSIT is the dominant protein encoded by the predicted splice variant Ecsit‐X4 in adult mouse hearts. This conclusion is supported by several lines of evidence: 2D gel electrophoresis (2‐DE) confirmed that the ECSIT antibody‐coupled protein is ≈42‐kDa, with a *pI* close to 8, which aligns with the Ecsit‐X4 isoform. Additionally, LC‐MS/MS identified five peptide sequences of the ≈42‐kDa ECSIT purified from the mitochondrial proteins of adult mouse heart tissues, matching the ECSIT‐X4 protein. The unique peptide sequence “tsmawagrttrmr” found in the ≈42‐kDa ECSIT distinguishes it from other proteins encoded by Ecsit splice variants. Notably, recent research has shown that human ECSIT is more unstable than its murine counterpart in heart tissues, and that ECSIT exists in mouse heart tissues as both a cytosolic (50‐kDa) and mitochondrial (45‐kDa) form.^[^ ^11^ ^]^ We found that the splicing junction of exon 9 gives rise to mouse Ecsit‐X4, which encodes a ≈42‐kDa ECSIT protein with a unique C‐terminal sequence, while sharing the mitochondrial‐targeting sequence (amino acids 1–48).

Increasing evidence has demonstrated that ECSIT is closely related to the occurrence and development of diseases, including Alzheimer's disease,^[^ ^10^ ^]^ embryonic development defects,^[^ ^12^ ^]^ ischemic stroke,^[^ ^21^ ^]^ fibrodysplasia ossificans progressiva (FOP) lesions,^[^ ^22^ ^]^ and tumorigenesis.^[^ ^23^ ^]^ ECSIT is also well recognized as a molecular link between oxidative stress, inflammation, and mitochondrial dysfunction.^[^ ^7^ ^]^ Xu et al. reported that in transgenic mice, where the murine Ecsit gene was replaced with the human ECSIT gene, low level of human ECSIT protein led to impaired oxidative phosphorylation and severe cardiac hypertrophy under physiological conditions. They emphasized that the observed effects in transgenic mice were attributed to reduced level of human ECSIT rather than functional differences between human ECSIT and murine Ecsit, and proposed the idea of a “threshold level” of Ecsit expression.^[^ ^11a^ ^]^ Another study, however, suggested that rather than a threshold level, the effects may be attributed to tissue‐specific cleavage of Ecsit and its integration into the fully assembled Complex I holoenzyme, as well as tissue‐specific differences in the Complex I assembly process, particularly in the stages controlled by ECSIT.^[^ ^11b^ ^]^ Importantly, studies regarding ECSIT isoform and its effects on pathological factors‐induced cardiac hypertrophy remain limited. We identified the previously unrecognized transcript variant Ecsit‐X4, and demonstrated that it is down‐regulated in response to pressure overload in hypertrophic mouse hearts and human samples. Additionally, through AAV9‐mediated overexpression of Ecsit‐X4 before or after TAC surgery, we confirmed that Ecsit‐X4 gene therapy significantly prevents pressure overload‐induced pathological cardiac hypertrophy. This study not only examined how the murine ~42‐kDa ECSIT is encoded, its tissue‐ and cell‐specific expression, and its localization, but also highlighted the significance of ECSIT‐X4 up‐regulation in pressure overload‐induced cardiac hypertrophy. To investigate the significant role of ECSIT‐X4 in mitigating pathological cardiac hypertrophy independently, we engineered cardiomyocyte‐specific Ecsit gene knockout mice and induced ECSIT‐X4 expression by injecting AAV9‐Ecsit‐X4 via the tail vein. Similarly, in vitro experiments were conducted by inducing Ecsit gene knockout in cardiomyocytes isolated from ECSIT^f/f^ mice using Adv‐Cre and additional overexpressing ECSIT‐X4 by transfecting Adv‐Flag‐Ecsit‐X4. Both of these methods demonstrated the novel role of ECSIT‐X4 in the prevention of pathological cardiac hypertrophy independently. To the best of our knowledge, this is the first study to report the ~42‐kDa ECSIT encoded by the transcript variant Ecsit‐X4 as a cardiac ECSIT isoform involved in pathological cardiac hypertrophy. Additionally, we have compared the functional differences between ECSIT‐X4 and the ~55‐kDa ECSIT isoform in Ang II‐treated NMCM‐Ecsit^cKO^. We found that the ~55‐kDa ECSIT isoform mediates NF‐κB signaling activation, mitochondrial dysfunction, and hypertrophy, whereas ECSIT‐X4 does not regulate NF‐κB signaling but instead exerts protective effects by maintaining mitochondrial function and inhibiting hypertrophy in Ang II‐treated NMCM‐Ecsit^cKO^. These data indicate ~55‐kDa ECSIT and ECSIT‐X4 play different roles and are also not influenced by other endogenous ECSIT isoform (Figure S8A–I, Supporting Information). Nonetheless, while the in vitro comparative studies suggest a potentially specific protective effect of ECSIT‐X4 against Ang II‐induced cardiomyocyte hypertrophy, a limitation remains in the lack of additional in vivo validation to definitively establish its targeted protective role in pressure overload‐induced cardiac hypertrophy in this study. In this context, more studies are needed to elucidate the regulatory mechanisms of Ecsit alternative splicing in the heart, including a thorough examination of the functional differences between its isoforms and the caution required when considering the targeting of specific isoforms for heart failure treatment.

Mitochondria are the core of aerobic respiration in cardiomyocytes, where ATP produced by oxidative phosphorylation, and it is a key factor in maintaining cardiac systolic and diastolic function.^[^ ^3^, ^24^ ^]^ This study found that endogenous ECSIT‐X4 expression is down‐regulated in mitochondria in hypertrophic mouse myocardium, and the expression of exogenous C‐terminal FLAG‐tagged ECSIT‐X4 results in autonomous mitochondrial localization in cultured cardiomyocytes. In addition, we demonstrated that ECSIT‐X4 increased the activity of mitochondrial complex I and up‐regulated the expression levels of key assembly proteins in hypertrophic cardiomyocytes. It has been shown that the activity and protein assembly of mitochondrial complexes II, III, IV, and V can be affected by the activity of complex I.^[^ ^25^ ^]^ This study found that ECSIT‐X4 overexpression not only significantly increased the expression of key assembly proteins of mitochondrial complex I but also directly or indirectly affected the assembly of the other four complexes. Mitochondrial dysfunction plays a pivotal role in accelerating the progression of various cardiovascular diseases, and our study demonstrated that ECSIT‐X4 provides protection against pressure overload‐induced myocardial hypertrophy and Ang II‐induced cardiomyocyte hypertrophy by enhancing mitochondrial function. This suggests that ECSIT‐X4 may exert broader protective effects in cardiovascular diseases characterized by mitochondrial dysfunction, potentially by preserving mitochondrial complex I integrity and function. However, while our data show a down‐regulation of ECSIT‐X4 expression in mitochondria in response to pressure overload, it remains unclear whether this alteration is purely a consequence of pressure overload itself or is a response to the mitochondrial dysfunction induced by such overload. If the latter is true, it would imply that ECSIT‐X4's protective role may not be limited to pressure overload‐induced hypertrophy but could also extend to other cardiovascular pathologies linked to mitochondrial dysfunction. Moreover, although NMCM‐Ecsit^cKO^ data clearly demonstrated that ECSIT‐X4 can exert an independent effect without relying on other isoforms, it is important to consider two factors: mitochondrial dysfunction resulting from Ecsit gene knockout and the presence of Ang II in vitro. Therefore, this protective effect may not be entirely specific to response to Ang II‐induced hypertrophic signaling pathway alone; rather, it is likely mediated by the restoration of mitochondrial function. In this sense, ECSIT‐X4's role in counteracting Ang II‐induced cardiomyocyte hypertrophy may also reflect a broader cellular response, particularly the normalization of mitochondrial energetics.

STAT3, in addition to its roles in the nucleus, has also been verified as a noncanonical regulator in mitochondrial function.^[^ ^26^ ^]^ Accumulating evidence suggests that mitochondrial STAT3 is involved in ischemic cardiomyopathy, post‐partum cardiopathy, cardiac hypertrophy, and heart failure.^[^ ^13^, ^26^, ^27^ ^]^ STAT3 has been shown to interact with ETC complex I, promoting ATP synthesis and reducing mROS in cardiac mitochondria.^[^ ^28^ ^]^ Aberrant changes in STAT3 protein expression and phosphorylation level at S727 contribute to mitochondrial dysfunction.^[^ ^17^ ^]^ In this study, we confirmed that ECSIT‐X4 interacts with STAT3 and increased its total protein and S727 phosphorylation level in mitochondria of cardiomyocytes, which supports mitochondria bioenergetics in hypertrophic cardiomyocytes. The use of a STAT3 inhibitor or suppression of its gene expression confirmed the role of ECSIT‐X4 in regulating mitochondrial OXPHOS function through STAT3. Although inhibiting STAT3 did not completely reverse the protective effects of ECSIT‐X4 overexpression—possibly due to incomplete gene deletion, insufficient STAT3 activity inhibition, or other ECSIT‐X4‐mediated molecular mechanisms—the data supported that ECSIT‐X4 regulates mitochondrial function, at least in part, through mitochondrial STAT3. Collectively, this study has unveiled a novel molecular mechanism involving the interaction between ECSIT‐X4 and STAT3 within mitochondria, which serves to mitigate energy deficits in hypertrophic cardiomyocytes and pathological cardiac hypertrophy.

## 4 Conclusion

In summary, this work addressed the important scientific question of whether the murine ~42‐kDa ECSIT is encoded by the transcript variant Ecsit‐X4, and highlights its critical role in suppressing pressure overload‐induced pathological cardiac hypertrophy and mitigating mitochondrial dysfunction through a novel pathway involving interaction with STAT3 to enhance its activity.

## 5 Experimental Section

### Human Tissue Sampling

Human myocardium samples used in this study were obtained during cardiac valve replacement surgeries or from organ donations. Written informed consent was obtained from patients or from the families of donors included in this research. The study was approved by the Institutional Review Board of the First Affiliated Hospital of Nanjing Medical University and adhered to the ethical guidelines of the 1975 Declaration of Helsinki. Detailed information is provided in Figure S1B (Supporting Information).

### Animals

The subjects in this study were male C57BL/6J mice. All mice were obtained from Center of Experimental Animals at Nanjing Medical University (NJMU) and housed in the Animal Laboratory Resource Facility of Nanjing Medical University. Ecsit^f/f^ mice^[^ ^23b^ ^]^ were generously provided by Professor Yang in the Department of Immunology, Key Laboratory of Immunological Environment and Disease, State Key Laboratory of Reproductive Medicine, Nanjing Medical University. Cardiomyocyte‐specific Ecsit knockout mice (Ecsit^cKO^) were generated by crossing Ecsit^f/f^ mice with Myh6‐Cre^ERT^ mice, followed by treatment with 50 mg kg^−1^ day^−1^ of tamoxifen for 5 days. The mice were maintained under controlled environmental conditions, including a 12‐h light/dark cycle, a room temperature of 22–24 °C, and humidity levels of 40–70%. They had ad libitum access to water and a standard chow diet. All investigations were conducted in accordance with the *Guidelines for the Care and Use of Laboratory Animals* published by the US National Institutes of Health (NIH Publication, 8th Edition, 2011). All aspects of animal care and experimental protocols were approved by the Nanjing Medical University Committee on Animal Care (Permit Number: IACUC‐2106014).

### Construction of Adenovirus (Adv) and Adeno‐Associated Virus 9 (AAV9)

The plasmid construct encoding the mouse Ecsit‐X4 gene (XM_011242539.4) was utilized to generate recombinant adenovirus (Adv). Adv‐GFP and Adv‐Flag‐Ecsit‐X4 under the control of the mouse cytomegalovirus (CMV) promoter were packaged by Genechem (Shanghai, China). A heart‐specific chicken cardiac troponin T (cTnT) promoter was used to drive the expression or suppression of Ecsit‐X4 or Stat3 in cardiomyocytes. A short hairpin RNA (shRNA) targeting Stat3 was synthesized with the following sequence: CGACTTTGATTTCAACTACAA (ShStat3). The scramble control sequence was: TTCTCCGAACGTGTCACGT. For in vitro experiments in cultured cardiomyocytes, cells were put on serum‐free medium and subsequently transfected with Adv‐GFP (MOI = 50) or Adv‐Flag‐Ecsit‐X4 (MOI = 50). For in vivo experiments in mice, AAV9‐GFP or AAV9‐Ecsit‐X4, or AAV9‐ShStat3 (3 × 10^11^ vg) was administered via tail vein injection.

### Mouse Model of Transverse Aortic Constriction (TAC)

A cardiac hypertrophy mouse model was established using transverse aortic constriction (TAC) following previously described procedures.^[^ ^29^ ^]^ Male mice, aged 6–10 weeks and weighing 20–25 g, were anesthetized with a mixture of isoflurane (1.5%) and oxygen (0.5 L min^−1^). The left thoracic cavity was opened to expose the aorta. A 27‐gauge needle was placed parallel to the transverse aorta, and the aorta was ligated using 5‐0 silk sutures between the first and second branches of the aortic arch. After ligation, the needle was removed, leaving the aorta constricted to the needle's diameter. For the sham control group, the same procedure was performed, except without the ligation step. Heart samples were harvested at 4 and 6 weeks after the surgical operation. The heart weight‐to‐body weight ratio (HW/BW) and lung weight‐to‐body weight ratio (LVW/BW) were calculated. Heart tissues were either snap‐frozen in liquid nitrogen and stored at −80 °C for RNA and protein analysis, or fixed in 4% paraformaldehyde in PBS for histological examination.

### Cell Culture, Gene Transfer, and Treatment

H9c2 cells, obtained from the American Type Culture Collection (ATCC), were cultured in Dulbecco's Modified Eagle Medium (DMEM, Invitrogen Corporation, USA) supplemented with 10% FBS, penicillin, and streptomycin. They were transfected with either Adv‐GFP (MOI = 50) or Adv‐Flag‐Ecsit‐X4 (MOI = 50) for 4 h. After transfection, the medium containing the adenovirus was replaced with DMEM supplemented with 10% FBS, and the cells were incubated for an additional 20 h. Prior to treatment with Ang II at a concentration of 1 µm, the cells were placed in serum‐free medium for 30 min. Ang II treatment was conducted 48 h post‐transfection.

Neonatal mouse cardiac myocytes (NMCMs) were isolated from 1 to 3‐day‐old C57BL/6J mice (obtained from Center of Experimental Animals, Nanjing Medical University) as previously described.^[^ ^29^, ^30^ ^]^ NMCMs were plated at a density of 1 × 10^6^ cells mL^−1^ and cultured in DMEM supplemented with penicillin, streptomycin, and 10% FBS. The cells were maintained under these conditions for the specified experiments.

Neonatal mouse cardiac myocytes (NMCMs) were isolated from 1 to 3‐day‐old Ecsit^f/f^ mice (NMCM‐Ecsit^f/f^) obtained from the Center of Experimental Animals, Nanjing Medical University. The cultivation method of NMCM‐Ecsit^f/f^ was described above. NMCM‐Ecsit^f/f^ cells were first incubated in serum‐free medium for 30 min and then transfected with Adv‐GFP or Adv‐Cre for 4 h. After transfection, the medium containing the adenovirus was replaced with DMEM supplemented with 10% FBS for 8 h. Subsequently, the cells were transfected with Adv‐GFP or Adv‐Flag‐Ecsit‐X4 for 4 h. Following this, the serum‐free medium was replaced with DMEM supplemented with 10% FBS for 20 h, after which Ang II stimulation was applied for 48 h.

### Plasmid and Si‐RNA Transfection

HEK293T cells, obtained from American‐type culture collection (ATCC), were cultured in DMEM supplemented with 10% FBS, penicillin, and streptomycin. Flag‐tagged Ecsit‐X4 were cloned from the mouse (8‐weeks old) heart cDNAs. Mouse expression plasmids encoding HA‐tagged Stat3 was purchased from Genechem (Shanghai, China). Mouse expression plasmids encoding Flag‐tagged ΔEcsit‐X4 (mutant with 110‐310 amino acid) and His‐tagged Stat3 were produced and purchased from Miaoling (Wuhan, China). Si‐RNA used for transfection in vitro was obtained from genepharma (Shanghai, China). Target sequence of Si‐Stat3: 5′‐GGCAUUCGGAAAGUAUUGUTT‐3′. For transient transfection, the cells were transduced with the indicated plasmids using Lipofectamine 3000 reagent (Invitrogen) according to the manufacturer's recommendations. In brief, when the cells reached 60–70% confluence, they were placed in Opti‐MEM and transfected with the plasmids using Lipofectamine 3000 reagent. After 6 h, the transfection mixture was replaced with DMEM‐supplemented 10% FBS.

### 2D Gel Electrophoresis (2‐DE) and Image Analysis

450 µg of total proteins were extracted from the heart tissue of 8‐week‐old mice. The ECSIT immunoprecipitates were separated by 2D gel electrophoresis (2‐DE) and SDS‐PAGE, followed by incubation of ECSIT antibody, and the signals were detected using the enhanced‐chemiluminescent (ECL) system (Pierce).

### Identification of ECSIT‐X4

Mitochondrial proteins were isolated from adult mouse hearts (8 weeks old). For identification of ECSIT‐X4, immunoprecipitation was performed using an anti‐ECSIT or anti‐IgG antibody, followed by electrophoresis and silver staining. Then the gel corresponding to the ~42‐kDa band was excised for liquid chromatography‐tandem mass spectrometry (LC‐MS/MS) analysis. The LC‐MS/MS was carried out by the Institute of Biophysics at the Chinese Academy of Sciences. Targeted proteins were digested with trypsin at a 1:50 ratio for 16 h, then purified using Pierce NeutrAvidin Plus UltraLink Resin (53151, ThermoFisher, USA). Peptides were separated using the NanoLC 1D Plus system. Eluting peptide cations were ionized by nanospray and analyzed on an LTQ‐Orbitrap XL mass spectrometer (Thermo Fisher, San Jose, USA). The MS/MS spectra were searched against the protein sequence of ECSIT‐X4.

### Western Blot

Protein extraction was performed from mouse heart tissues and cultured cells, respectively. Immunoblots were conducted as previously described.^[^ ^29^ ^]^ Briefly, Western blot analyses were carried out using commercially available antibodies: anti‐ECSIT (ab21288, abcam), anti‐α‐actinin (ab137346, abcam), anti‐VDAC1 (ab235143, abcam), anti‐GST (ab138491, abcam), anti‐OXPHOXS (ab110413, abcam), anti‐NDUFAF1 (ab79826, abcam), anti‐cTnT (ab8295, abcam), anti‐NDUFA13 (ab110240, abcam), anti‐NDUFB8 (ab192878, abcam), anti‐STAT3 (12640, CST), anti‐p (S727) STAT3 (49081, CST), anti‐GAPDH (AF0006, Beyotime), anti‐Tubulin (AT819, Beyotime), followed by incubation with peroxidase‐conjugated secondary antibodies. The signals were detected using the enhanced‐chemiluminescent (ECL) system (Pierce) and quantified by scanning densitometry with Image Lab analysis system. Tubulin, GAPDH, and VDAC1 served as the loading control. The results from each experimental group were expressed as relative integrated intensity compared to the control group measured at the same time.

### Real‐Time PCR

Total RNA was extracted as previously described,^[^ ^29^ ^]^ and then converted into cDNA using PrimeScriptTM RT Reagent kit with gDNA eraser (Takara RR047A, Japan). Quantitative RT‐PCR reactions were performed using the ChamQTM SYBR Color qPCR Master Mix (Vazyme, China) and QuantStudio (TM) 6 Flex System (Applied Biosystems, USA). Hypoxanthine‐guanine phosphoribosyltransferase (Hprt) was used as the internal reference for each sample. The primer sequences are shown in Table S1 (Supporting Information).

### Echocardiography

Cardiac function was assessed using a Vevo 2100 High‐Resolution Micro‐Ultrasound System (FUJIFILM Visual Sonics Inc.). Mice were anesthetized with a mixture of isoflurane (1.5%) and oxygen (0.5 L min^−1^) and placed in the supine position on a heating table. 2D guided left ventricular M‐mode tracings were obtained at the level of the papillary muscle in a short‐axis view to measure parameters such as left ventricular posterior wall thickness (LVPW), interventricular septum thickness (IVS), left ventricular internal dimension (LVID), ejection fraction (EF%), and fractional shortening (FS%). All measurements were performed by a blinded researcher to minimize bias and were averaged over five consecutive cardiac cycles.

### GST‐Pull Down Assay

Bacterial‐expressed GST‐ECSIT‐X4 or control GST, bound to MagneGST Glutathione Particles, was incubated with purified His‐tagged Stat3 protein for 3 h at 4 °C using the MagneGST Pull‐Down System (Thermo Scientific, Rockford, USA) according to the manufacturer's instructions. The washed complexes were eluted by boiling in SDS sample buffer, separated by SDS‐PAGE, and the interactions were analyzed by Western blot.

### Isolation of Mitochondria

Fresh heart tissue (60 mg) or 5 × 10^6^ to 2 × 10^7^ cells were prepared for extraction. The heart tissues or cells were lysed using ice‐cold lysis buffer with protease inhibitor solution, following standard protocols. The lysate was centrifuged at 1000 × g for 10 min at 4 °C, and cytosolic proteins were collected from the supernatant. The remaining cell pellet was resuspended in disruption buffer and thoroughly disrupted using a blunt‐ended needle and syringe. After a second centrifugation at 1000 × g for 10 min at 4 °C, the supernatant was transferred to a clean 1.5 mL tube. The supernatant was then centrifuged at 6000 × g for 10 min at 4 °C. The resulting pellet, containing mitochondria, was purified using mitochondria purification buffer and centrifuged at 14 000 × g for 15 min at 4 °C to obtain isolated mitochondria.

### Mitochondria Complex I Activity, mROS, and ATP Analysis

Isolated mitochondria were prepared and measured using the MitoCheckComplex I Activity Assay (Cayman, USA). The procedure was performed according to the manufacturer's instructions. The rate of NADH oxidation was measured by the decrease in absorbance at 340 nm, and it is proportional to the activity of complex I. Plot the data as absorbance (Y‐axis) versus time (X‐axis) and calculate the slope for the linear portion of the curve. Determine the percentage of activity relative to the vehicle control using the equation indicted below: Complex I activity (%) = (Rate of sample wells/Rate of vehicle control) ×100%.

Cells were washed with pre‐cooled PBS and then incubated in the dark with 5 µm MitoSOX (Life Techonolgies, USA) for 15 min, followed by incubation of Hoechst for 10 min at 37 °C. Fluorescence measurements were performed using the microscope (OLYMPUS TH4‐200, Japan), and five random fields of view were selected for photography. The content of mROS were quantified using Image J software. Intracellular ATP concentration was measured with an ATP Assay Kit (Beyotime, China).

### Histological Examination

Mouse and human hearts were collected, and immersion fixed in 4% buffered paraformaldehyde. The heart tissues were then embedded in paraffin and cut at 5 µm. Briefly, all the mouse and human heart tissues were counter‐stained with hematoxylin and eosin (H&E). Mouse heart sections also incubated with Masson's Trichrome Stain Kit (Solarbio, China).

### Immunoprecipitation‐Mass Spectrum (IP‐MS)

NMCMs isolated from Ecsit^f/f^ mice were cultivated on 10‐cm plates and subsequently transfected with Adv‐Flag‐Ecsit‐X4. Mitochondria isolated from the NMCMs were lysed and immunoprecipitated with either anti‐IgG or anti‐Flag antibody. Flag‐labeled immunoprecipitates were visualized by Coomassie blue staining and subjected to LC‐MS/MS analysis. The resulting MS/MS spectra were analyzed against the UniProt mouse proteome database using SequestHT.

### Co‐Immunoprecipitation

Cells were harvested and lysed as previously described.^[^ ^29^ ^]^ The lysates were incubated overnight at 4 °C with either anti‐Flag (Sigma, USA) or anti‐STAT3 (CST, USA) antibodies. Next, 30 µL of protein G agarose beads (Cytiva) were added and co‐incubated with the immune complexes for 4 h at 4 °C. After washing three times with cold wash buffer and once with lysis buffer, the immunoprecipitates were resuspended in 30 µL of lysis buffer and analyzed via Western blot to detect the indicated target proteins.

### Immunofluorescence

Heart tissue sections were dewaxed and rehydrated, while cells were fixed in 4% paraformaldehyde. After washing, the heart tissue sections or cells were permeabilized with 0.1% Triton X‐100 and blocked with 5% normal goat serum (BioGenex, Fremont, CA). Samples were then stained with one or more antibodies according to the manufacturer's instructions, followed by DAPI (Invitrogen) staining. Subsequently, they were incubated with Alexa Fluor 488, 594, or 633‐conjugated secondary antibodies (1:500) (Life Technologies, Carlsbad, CA) at 37 °C for 90 min. Images from five randomly selected fields per group were captured using a confocal microscope (Zeiss, Germany).

### Statistics

All data were presented as means ± SD. Statistical analysis was performed using GraphPad Prism 8.0. For comparisons between two groups, a two‐tailed unpaired Student's *t*‐test was conducted in the condition of homogeneity of variance (*p* > 0.1). When comparing more than two groups, one‐way or two‐way analysis of variance (ANOVA) corrected by the post hoc Turkey's test was carried out. In all cases, *p <* 0.05 was considered statistically significant. More information about statistical details including sample size (*n*) for each statistical analysis is indicated in the figure legends.

## Conflict of Interest

The authors declare no conflict of interest.

## Author Contributions

Y.H.L., S.Y., J.T.L., X.L., and T.T.T. were involved in the conceptualization of the study. X.L. and T.T.T. designed and performed the experiments. Y.H.L. supervised the project. X.L. and T.T.T. analyzed the data, prepared the figures, and edited the manuscript. Y.H.L., S.Y., J.T.L., X.L., T.T.T., H.L.S., Y.C., Y.F.S., P.X.S., L.L.Q., L.L., G.Q.Z., Q.C., and C.F.L. were involved in the review of the manuscript. Y.H.L., J.T.L., and X.L. acquired funding for the studies.

## Supporting information



Supporting Information

## Data Availability

The data that support the findings of this study are available from the corresponding author upon reasonable request.
