# Supplementary material for: ECSIT‐X4 is Required for Preventing Pressure Overload‐Induced Cardiac Hypertrophy via Regulating Mitochondrial STAT3
Source: Adv Sci (Weinh). 2025 Jan 2;12(8):2414358. doi: 10.1002/advs.202414358 (PMC11848529; doi:10.1002/advs.202414358)
Supplement: Supplementary file 1 — Supporting Information [file ADVS-12-2414358-s001.docx]

**SUPPLEMENTARY MATERIALS**

**ECSIT-X4 is Required for Preventing** **Pressure Overload-Induced Cardiac Hypertrophy via Regulating Mitochondrial STAT3**

Xia Lu^1,2^†, Tingting Tong^1^†, Haoliang Sun^3^, Yi Chen^1^, Yongfeng Shao^3^, Pengxi Shi^1^, Linli Que^1^, Li Liu^4^, Guoqing Zhu^1^, Qi Chen^1^, Chuanfu Li^5^, Jiantao Li^1*^, Shuo Yang^6*^, Yuehua Li^1*^

^1^Key Laboratory of Targeted Intervention of Cardiovascular Disease, Collaborative Innovation Center for Cardiovascular Disease Translational Medicine, School of Basic Medical Sciences, Nanjing Medical University, Nanjing, Jiangsu, 211166, China

^2^Department of Cardiology, Shanghai Sixth People’s Hospital Affiliated to Shanghai Jiao Tong University School of Medicine, Shanghai 200233, China

^3^Department of Cardiovascular Surgery, The First Affiliated Hospital of Nanjing Medical University, Nanjing, Jiangsu,210029, China

^4^Department of Geriatrics, the First Affiliated Hospital of Nanjing Medical University, Nanjing ,Jiangsu, 210029, China

^5^Department of Surgery, East Tennessee State University, Campus Box 70575, Johnson City, TN 37614-0575, USA

^6^Department of Immunology, Key Laboratory of Immunological Environment and Disease, State Key Laboratory of Reproductive Medicine, Nanjing Medical University, Nanjing, Jiangsu, 211166, China

†These authors contributed equally to this work.

*****Corresponding authors:

Yuehua Li, MD, Ph. D.

Professor in the Department of Pathophysiology

Nanjing Medical University

Nanjing, China, 211166

Email: [yhli@njmu.edu.cn](mailto:yhli@njmu.edu.cn);

Shuo Yang, MD, Ph. D.

Professor in the Department of Immunology

Nanjing Medical University

Nanjing, China, 211166

Email: [shuoyang01@njmu.edu.cn](mailto:shuoyang01@njmu.edu.cn);

Jiantao Li, Ph. D.

Associate Professor in the Department of Pathophysiology

Nanjing Medical University

Nanjing, China, 211166

Email: [ljt@njmu.edu.cn](mailto:ljt@njmu.edu.cn);

**Table S1. Primer sequences for real-time-PCR**

| Genes | Primer sequences | |
| --- | --- | --- |
| Mouse Ecsit-X4 | Forward  Reverse | CCGCCACAACCCATCCAG  CGCCAAACGGGGACAACT |
| Mouse ANP | Forward  Reverse | GAGAAGATGCCGGTAGAAGA  GCACTGCCGTCTCTCAGA |
| Mouse BNP | Forward  Reverse | GCTTTGGGCAGAAGATAGA  AGTTTGTGCTGGAAGATAA |
| Mouse β-MHC | Forward  Reverse | CTGGCACCGTGGACTA  GCTTGAGGGAGGACTTCTGG |
| Mouse Collagen I | Forward  Reverse | TCGTGACCGTGACCTTG  GAGGCACAGACGGCTGAGTAG |
| Mouse Collagen III | Forward  Reverse | CACCTTGGTCAGTCC  ATTCCTCCCACTCCAGACTTG |
| Mouse HPRT | Forward  Reverse | GTTGGGCTTACCTCACTGCT  TCATCGCTAATCACGACGCT |
| Rat ANP | Forward  Reverse | GAGTGAGCCGAGACAGCAA  TCTGAGACGGGTTGACTTCC |
| Rat BNP | Forward  Reverse | GCTTTGGGCAGAAGATAGA  CAAGTTTGTGCTGGAAGATAA |
| Rat β-MHC | Forward  Reverse | TGCTGGCACCGTGGACTA  TGCTGGCACCGTGGACTA |
| Rat HPRT | Forward  Reverse | GCTGAAGATTTGGAAAAGGTGT  ACAGAGGGCCACAATGTGAT |

**
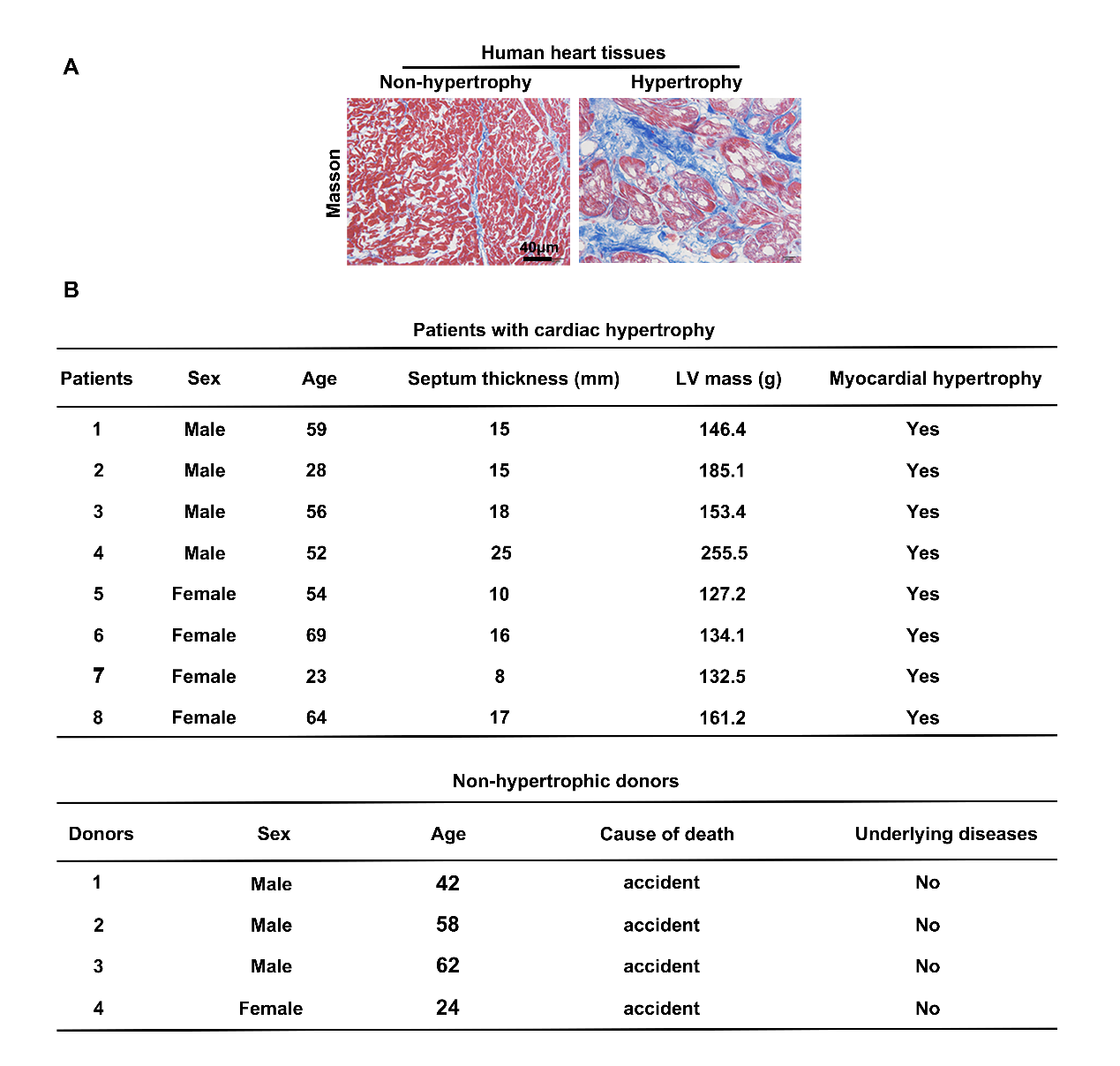
**

**Figure S****1. Clinical information of hypertrophic patients and non-hypertrophic donors.** A) Masson’s trichrome staining was used to assess myocardial interstitial collagen in human heart tissues. Scale bar = 40 μm. *n* = 4 to 8 samples per group. B) Human hypertrophic myocardium samples were obtained during cardiac valve replacement surgical procedures based on echocardiographic finding. Clinical information of the patients and the non-hypertrophic donors were presented in the table.


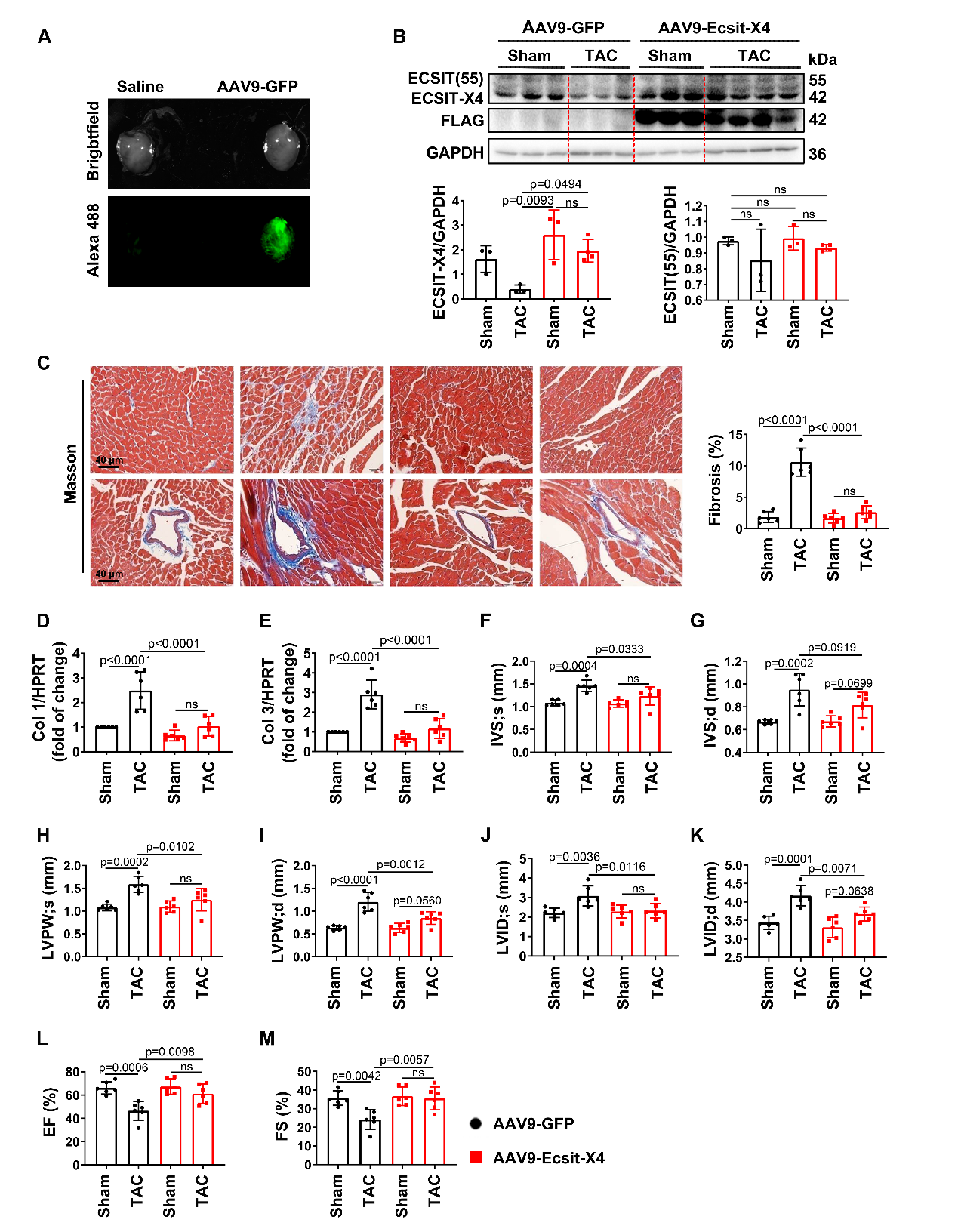
 **Figure S2. Overexpression of Ecsit-X4 before TAC surgery improves cardiac fibrosis and cardiac dysfunction.** A) Fluorescence of GFP in mouse hearts. B) Representative Western blot and statistical result of ECSIT-X4 in mice hearts. *n* = 3, 3, 3, 4, respectively. C) Masson’s trichrome staining was performed to detect myocardial collagen in mouse hearts. Scale bar = 40 μm. Statistical results of fibrosis was shown. D, E) The mRNA levels of Collagen I (Col 1) and Collagen III (Col 3) were detected by qRT-PCR. All were normalized to HPRT. F-M) Quantification of echocardiographic parameters: interventricular septum systolic dimension (IVS; s), interventricular septum diastolic dimension (IVS; d), left ventricle posterior wall thickness systole (LVPW; s), left ventricle posterior wall thickness diastole (LVPW; d), left ventricle internal dimension systole (LVID; s), left ventricular internal dimension diastole (LVID; d), ejection fraction (EF%), and fractional shortening (FS%). *n* = 6 mice per group. Data were presented as mean ± SD, *p*-values were determined by one-way ANOVA corrected by the post hoc Turkey’s test. *p <* 0.05 was considered statistically significant.

**
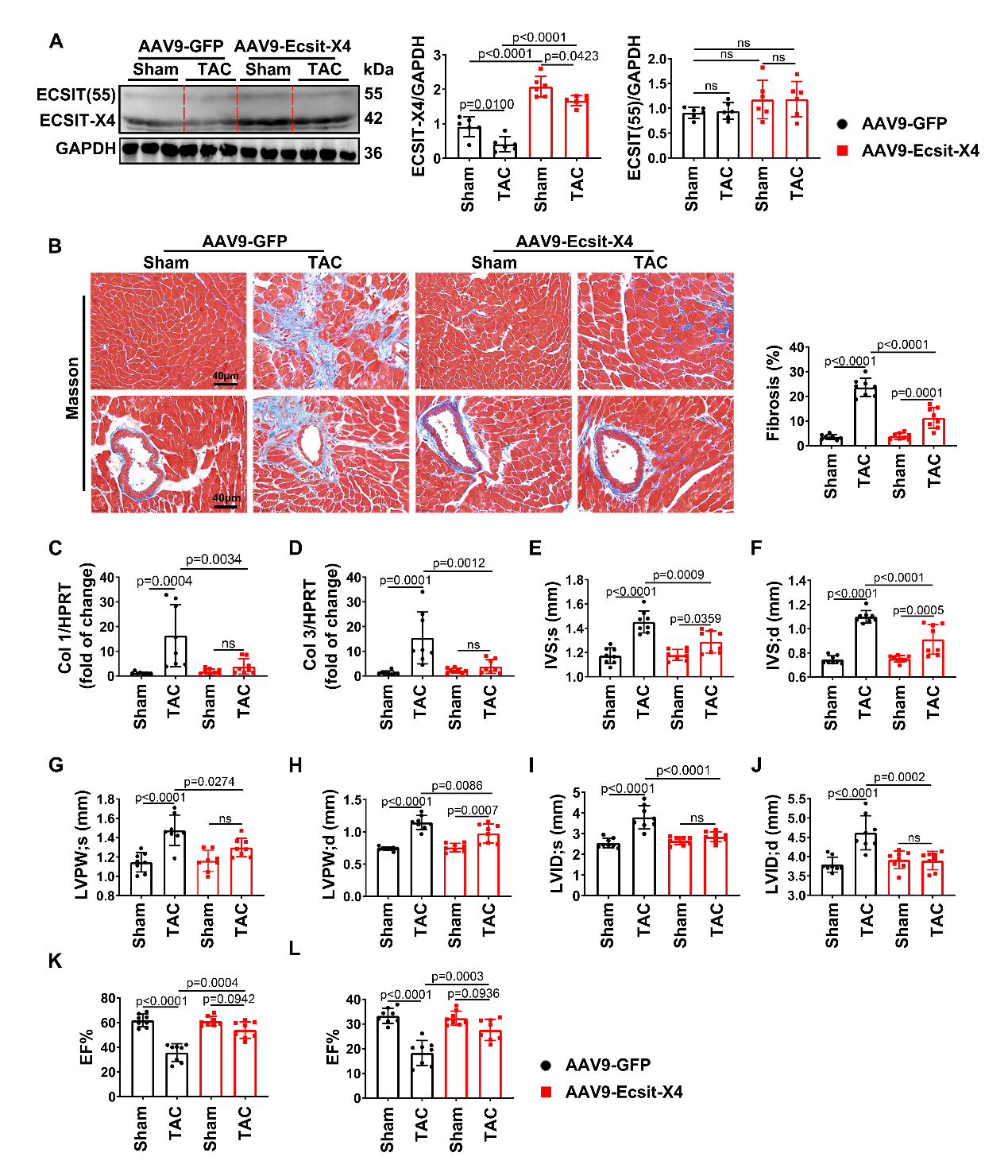
**

**Fig. S3. Overexpression of Ecsit-X4 after TAC surgery improves cardiac fibrosis and cardiac dysfunction.** A) Representative Western blot and statistical result of ECSIT-X4 in mice hearts. *n* = 6 mice per group. B) Masson’s trichrome staining was performed to detect myocardial collagen of mice hearts. Statistical result of fibrosis was presented**.** Scale bar = 40 μm. C, D) The mRNA levels of Collagen I (Col 1) and Collagen III (Col 3) were detected by qRT-PCR. All were normalized to HPRT. E-L) Quantification of echocardiographic parameters: IVS; s, IVS; d, LVPW; s, LVPW; d, LVID; s, LVID; d, EF%, and FS%. *n* = 8 mice per group. Data were presented as mean ± SD, *p*-values were determined by one-way ANOVA corrected by the post hoc Turkey’s test. *p <* 0.05 was considered statistically significant.


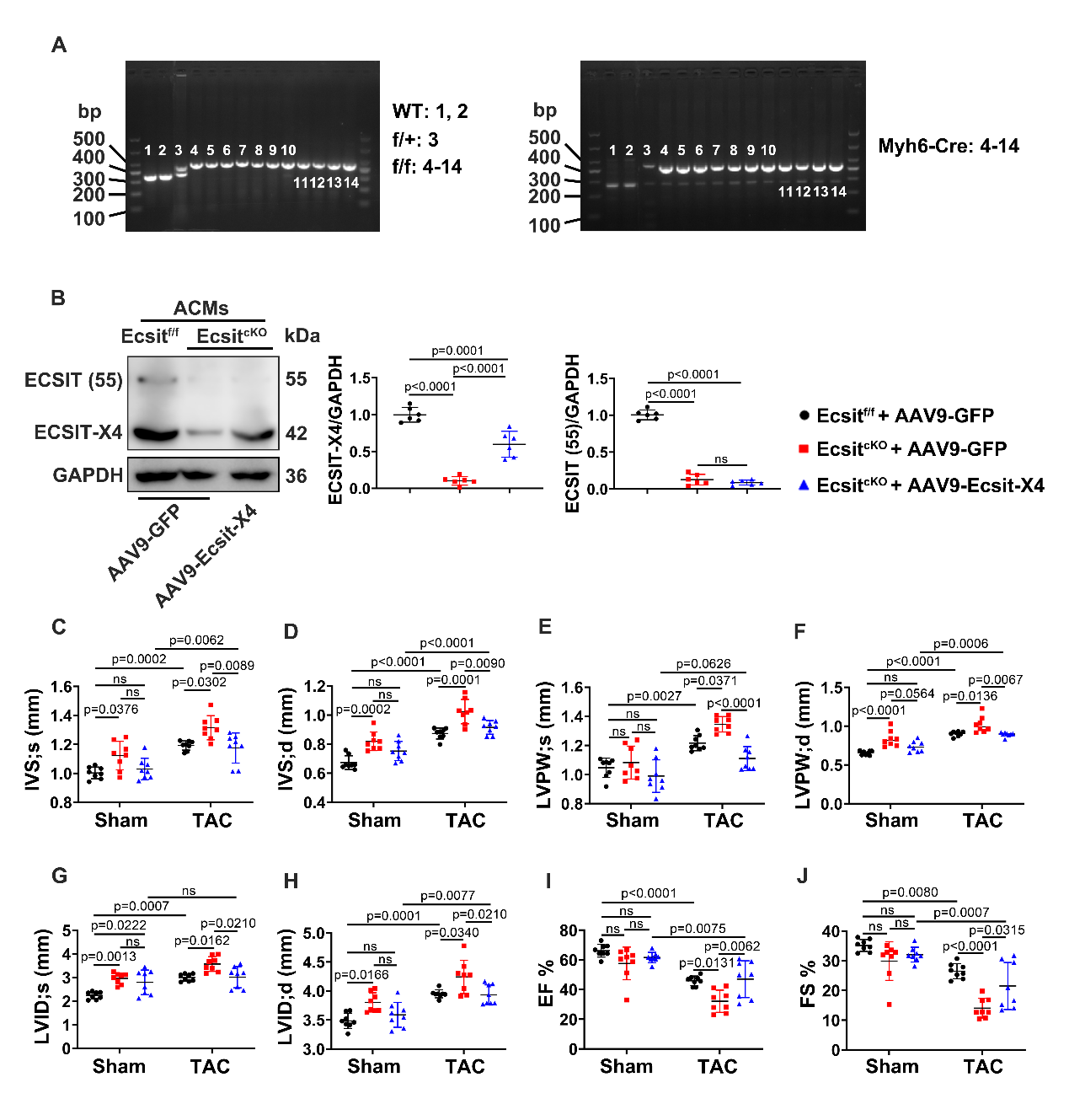
 **Figure S4. Compensation of Ecsit-X4 in Ecsit^cKO^ mice hearts improves pressure overload-induced cardiac dysfunction.** A) PCR analysis of DNA isolated from mouse tails. Primer sequence: 5’loxp-F CCCTGTAGCTCCAATGTGCTCAGAC; 5’loxp-R AGCAACCACCCTATTTGTGGACACC. Myh6-Cre-F ATACCGGAGATCATGCAAGC; Myh6-Cre-R AGGTGGACCTGATCATGGAG. The heterozygous Myh6-Cre^ERT^ mice were crossed with Ecsit^f/f^ mice. Heterozygous F1 mice with Cre recombinase were selected for further breeding. The homozygous F2 mice with Cre recombinase (the mice numbered 4-14) were the cardiomyocyte-specific Ecsit knockout mice needed for the experiment. Heterozygous Ecsit^f/+^ mouse (the mice numbered 3); Wild type mice (the mice numbered 1-2). B) Representative Western blot and statistical result of ECSIT-X4 in cardiomyocytes isolated from mouse hearts. Adult cardiomyocytes (ACMs). *n* = 6 mice per group. C-J) Quantification of echocardiographic parameters: IVS; s, IVS; d, LVPW; s, LVPW; d, LVID; s, LVID; d, EF%, and FS%. *n* = 8 mice per group. Data were presented as mean ± SD, *p*-values were determined by two-way ANOVA corrected by the post hoc Turkey’s test. *p <* 0.05 was considered statistically significant.

**
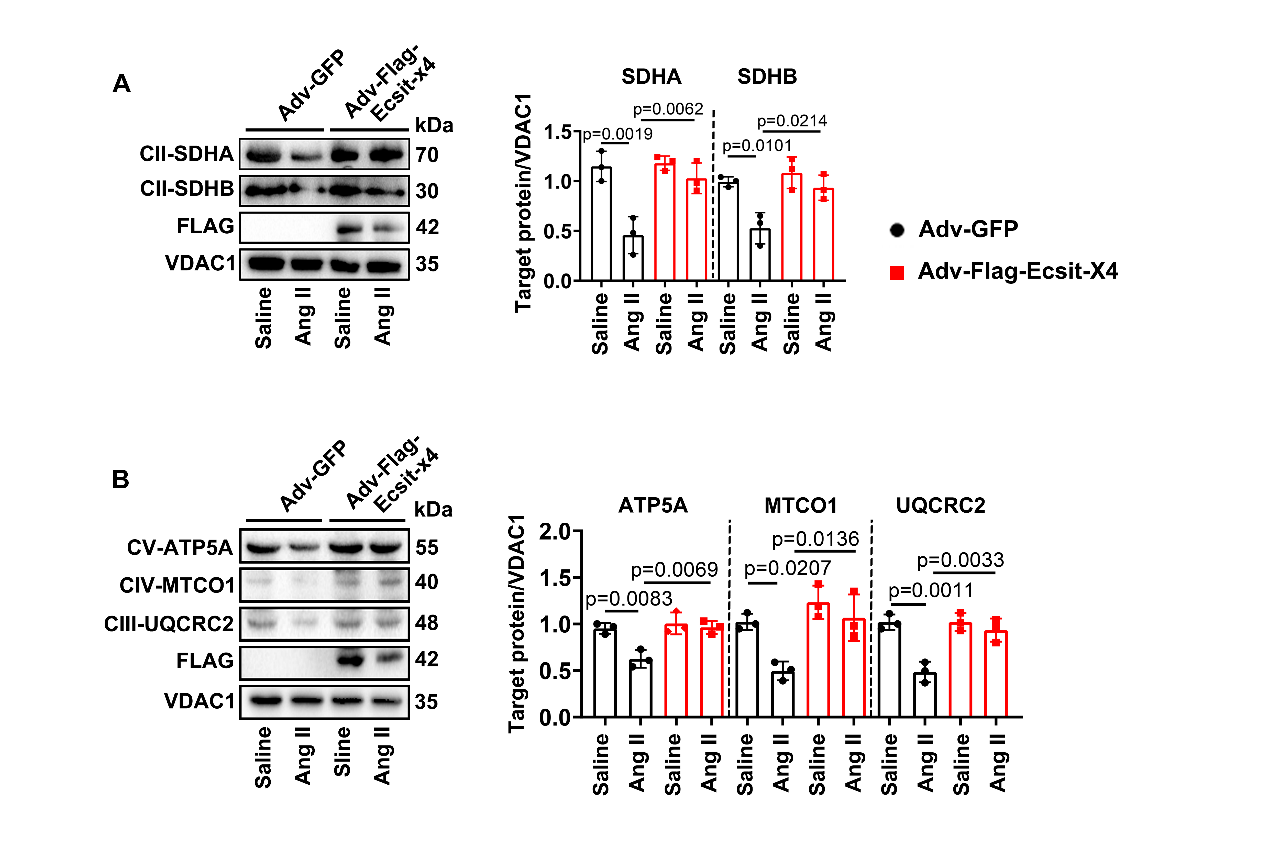
Figure S5. Overexpression of Ecsit-X4 enhances another four mitochondrial complexes in Ang II-induced hypertrophic cardiomyocytes.** A, B) Representative Western blots and statistical results of SDHA, SDHB, ATP5A, MTCO1, and UQCRC2 in mitochondria of H9c2 cells. *n* = 3 independent experiments. Data were presented as mean ± SD, *p*-values were determined by one-way ANOVA corrected by the post hoc Turkey’s test. *p <* 0.05 was considered statistically significant.

**
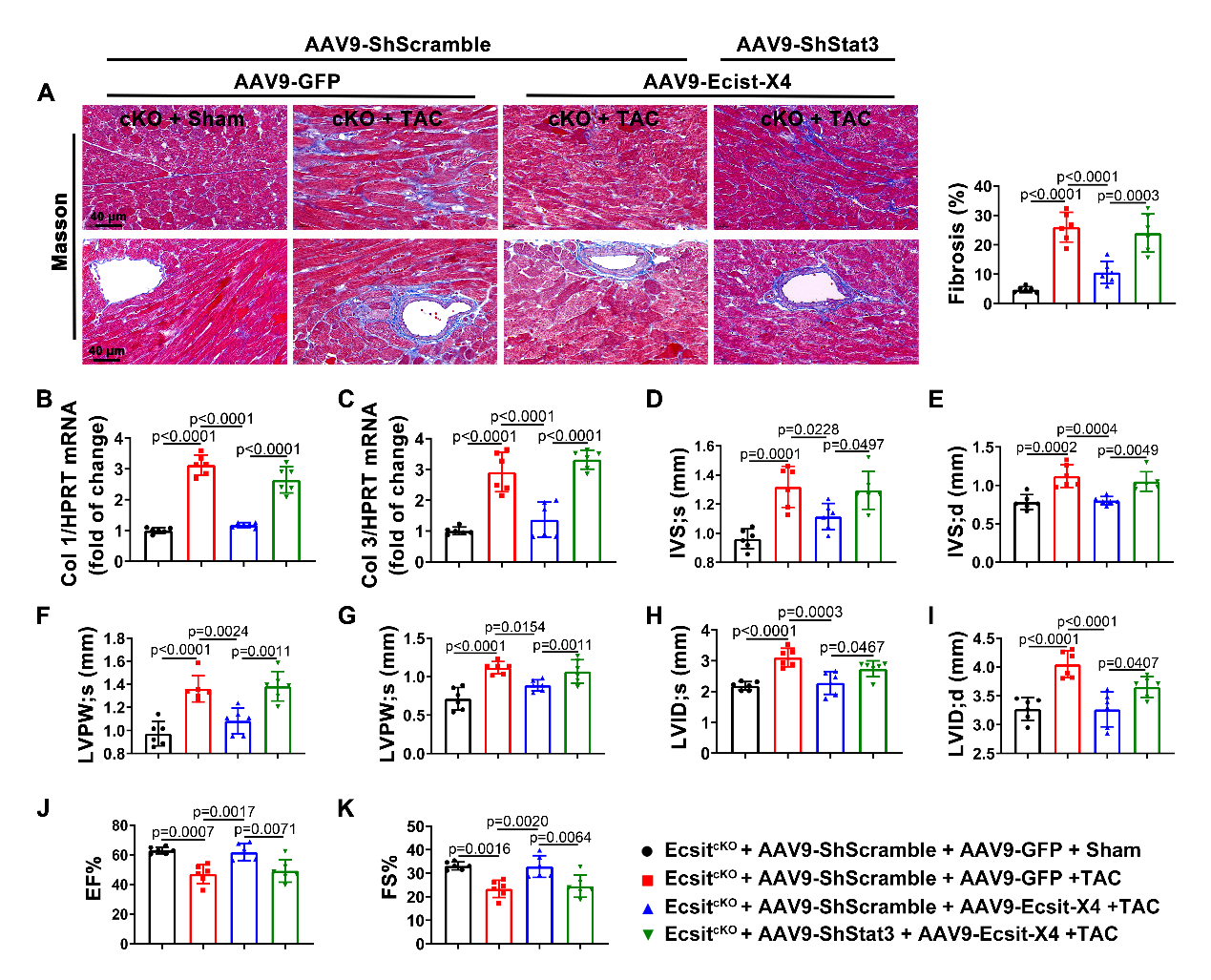
**

**Figure S6. ECSIT-X4 alleviates cardiac fibrosis and function through STAT3 in hypertrophic Ecsit^cKO^ mice.** A) Masson’s trichrome staining was performed to detect myocardial collagen of mouse hearts. Statistical result of fibrosis was presented**.** Scale bar = 40 μm. B, C) The mRNA levels of Collagen I (Col 1) and Collagen III (Col 3) were detected by qRT-PCR. All were normalized to HPRT. D-K) Quantification of echocardiographic parameters: IVS; s, IVS; d, LVPW; s, LVPW; d, LVID; s, LVID; d, EF%, and FS%. *n* = 6 mice per group. Data were presented as mean ± SD, *p*-values were determined by one-way ANOVA corrected by the post hoc Turkey’s test. *p <* 0.05 was considered statistically significant.


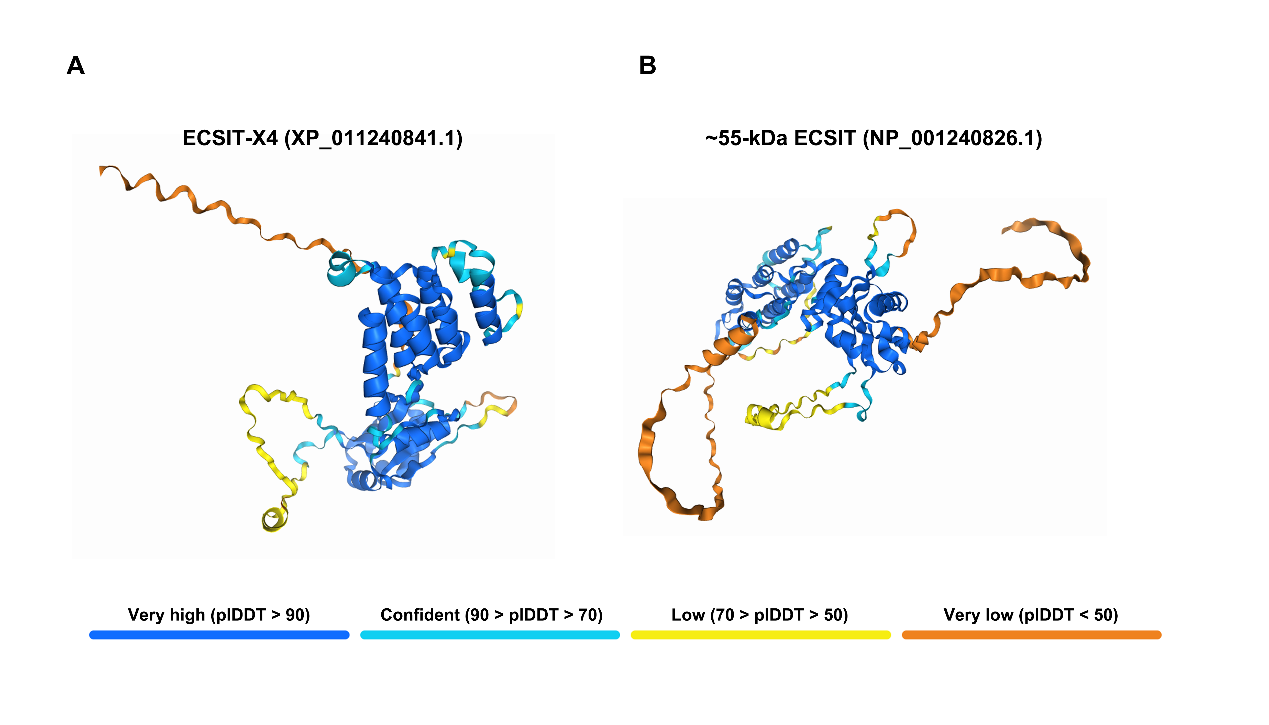


**Figure S7.** **The protein structure of ECSIT-X4 and ≈55-kDa ECSIT were build using AlphaFold2.** A, B) ECSIT-X4 (XP_011240841.1) has a compact structure with numerous stable helical regions (darker blue, pLDDT > 90). In contrast, ≈55-kDa ECSIT (NP_001240826.1) has a more dispersed and extended structure, particularly in its lower-confidence terminal region (orange, pLDDT < 50). While ECSIT-X4’s central part shows high confidence and stability, ≈55-kDa ECSIT contains more low-confidence areas (pLDDT < 70), indicating weaker structural integrity.


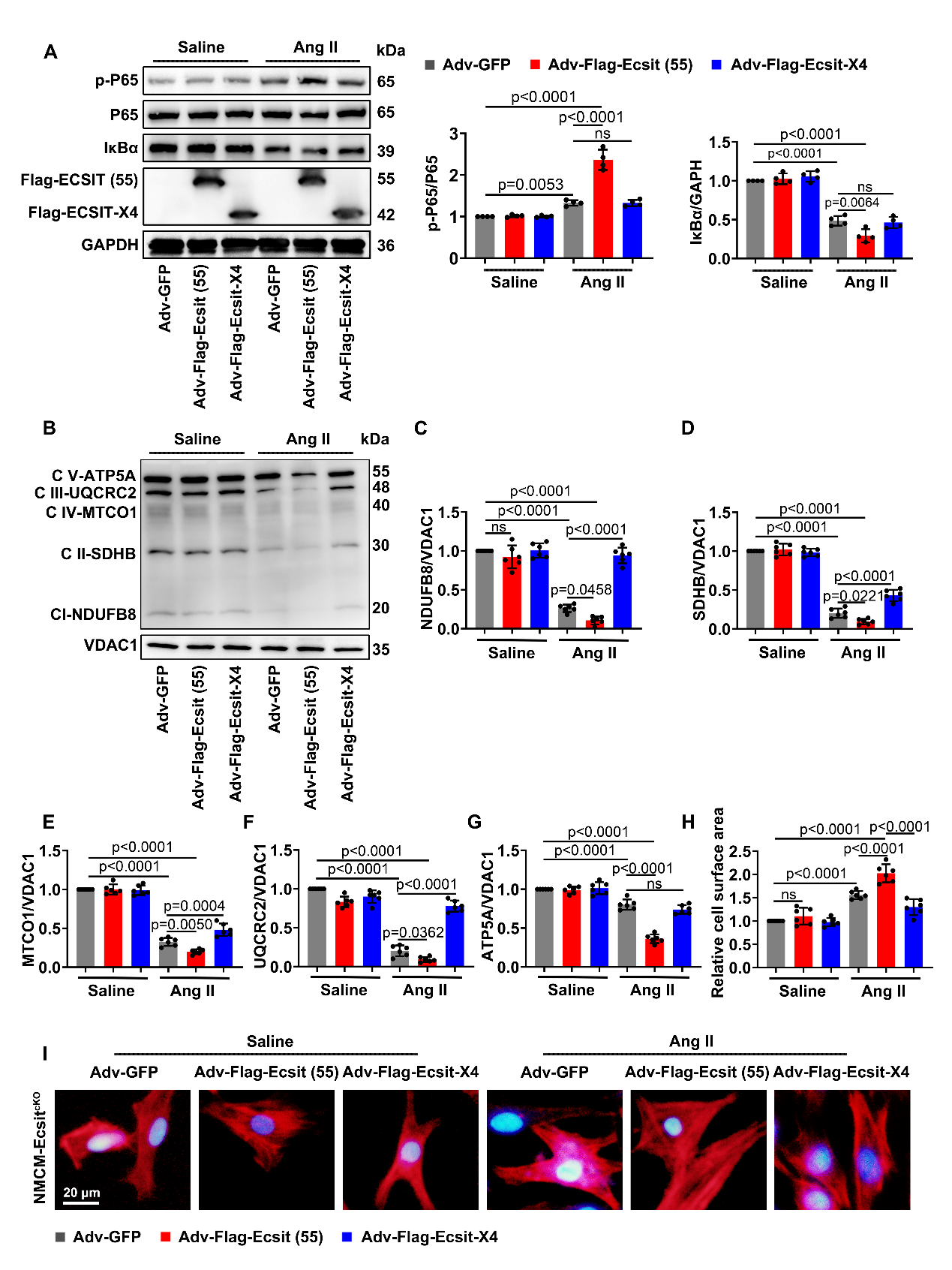


**Figure S8.** **The ≈55-kDa ECSIT isoform mediates NF-κB activation, mitochondrial dysfunction, and promotes cardiomyocyte hypertrophy, whereas ECSIT-X4 does not regulate NF-κB signaling but instead exerts protective effects.** A) NMCM-Ecsit^cKO^ transfected with Adv-GFP or Adv-Flag-Ecsit-(55) or Adv-Flag-Ecsit-X4 for 24 hours before they were treated with Saline or Ang II (1 μM) for 48 hours. Representative Western blots and statistical results of p-P65 and IκBα were shown. *n* = 4 independent experiments. B-G) Representative Western blots and statistical results of the ETC complex components. *n* = 6 independent experiments. H, I) Morphology of NMCM-Ecsit^cKO^ stained with α-actinin (red), with nuclei counterstained using DAPI (blue). Statistical result of cell surface areas was shown. Scale bar = 20 μm. *n* = 6 independent experiments. Data were presented as mean ± SD, *p*-values were determined by one-way ANOVA corrected by the post hoc Turkey’s test. *p <* 0.05 was considered statistically significant.
